# Palladium Catalysts Supported in Microporous Phosphine Polymer Networks

**DOI:** 10.3390/polym15204143

**Published:** 2023-10-19

**Authors:** Noelia Esteban, Miguel Claros, Cristina Álvarez, Ángel E. Lozano, Camino Bartolomé, Jesús M. Martínez-Ilarduya, Jesús A. Miguel

**Affiliations:** 1 IU CINQUIMA, School of Sciences, University of Valladolid, Paseo Belén 5, E-47011 Valladolid, Spain; isabelnoelia.esteban@uva.es (N.E.); m.claroscasielles@uva.nl (M.C.); cristina.alvarez@ictp.csic.es (C.Á.); lozano@ictp.csic.es (Á.E.L.); jmi@qi.uva.es (J.M.M.-I.); 2 SMAP, UA-UVA_CSIC, Associated Research Unit to CSIC, School of Sciences, University of Valladolid, Paseo Belén 7, E-47011 Valladolid, Spain; 3 Institute of Polymer Science and Technology, ICTP-CSIC, Juan de la Cierva 3, E-28006 Madrid, Spain

**Keywords:** knitting, phosphine-based POPs, palladium catalyst, Suzuki–Miyaura

## Abstract

A new set of microporous organic polymers (POPs) containing diphosphine derivatives synthesized by knitting via Friedel–Crafts has been attained. These amorphous three-dimensional materials have been prepared by utilizing diphosphines, 1,3,5-triphenylbenzene, and biphenyl as nucleophile aromatic groups, dimethoxymethane as the electrophilic linker, and FeCl_3_ as a promoting catalyst. These polymer networks display moderate thermal stability and high microporosity, boasting BET surface areas above 760 m^2^/g. They are capable of coordinating with palladium acetate, using the phosphine derivative as an anchoring center, and have proven to be highly efficient catalysts in Suzuki–Miyaura coupling reactions involving bromo- and chloroarenes under environmentally friendly (using water and ethanol as solvents) and aerobic conditions. These supported catalysts have achieved excellent turnover numbers (TON) and turnover frequencies (TOF), while maintaining good recyclability without significant loss of activity or Pd leaching after five consecutive reaction cycles.

## 1. Introduction

Porous organic polymers, POPs, have showed high efficiency as heterogeneous catalysts due to properties such as high microporosity with tunable pore volume, high specific surface area, the possibility to incorporate high catalyst loadings or excellent thermal and chemical stability. In recent years, the inclusion of catalysts confined in very small pores has resulted in materials with excellent reaction yields, high turnover number (TON) and turnover frequency (TOF), high regioselectivities, high recyclability and low metallic contamination in the products obtained [1,2,3,4,5,6,7,8]. These POPs can be synthesized by multiple methodologies [9]. Braiding or knitting is a versatile and powerful methodology to obtain hyper-crosslinked polymers, HCPs, from the assembly of aromatic monomers with methylene groups, or other linkers, via a Friedel–Crafts reaction promoted by FeCl_3_ or AlCl_3_. In this methodology, an electrophile, which acts as linker, one or more aromatic species that will act as nucleophiles, and a Lewis acid are required to achieve the desired hyper-crosslinking. By choosing the right combination of monomers (trifunctional, phosphine, and BP), it would be possible to obtain a porous polymeric network with phosphine active centers that could be used as efficient catalysts in different synthetic processes. Also, the use of other metals would extend their use in other organic reactions of high industrial importance [10,11,12,13,14,15,16,17,18]. Phosphine-based POP systems have been recently developed and used as an alternative to analogous molecular catalytic systems in different transformations of interest [19]. Furthermore, if phosphine-based POPs are designed correctly and a suitable metal is chosen, the steric and electronic environment around the coordinated metal center can be modulated, which will have an impact on the reactivity and specificity of the catalyst toward organic transformations. Lai’s group has published the direct polymerization of [Pd(PPh_3_)_4_] and benzene [2]. Song’s group has prepared materials by the knitting technique copolymerizing 1,1′-bis(diphenylphosphino)ferrocene and biphenyl, obtaining an efficient catalyst for the reduction of 4-nitrophenol, where the ferrocenyl units remain unchanged [3]. Recently, the preparation of polymers that maintain the molecular structure of the starting ruthenium or gold complexes with phosphine ligands and that behave as active catalysts in reactions of formic acid dehydrogenation [4], imination of alcohols [10], or hydration of alkynes [20] has been described. Two different synthetic methods have been used to obtain POPs from PPh_3_ (TPP) in the presence of Lewis acids (FeCl_3_ or AlCl_3_): the Scholl reaction [21] and the knitting method [22], in which dimethoxymethane, DMM, is used as an electrophile providing methylene groups as spacers between the aromatic fragments.

In the recent years, following the synthesis methodology described by Zolotukhin et al. [23,24,25], our research group has developed, using trifunctional aromatic molecules with a specific symmetry (triptycene, TRP, or 1,3,5-triphenylbenzene, 135TPB), new rigid, amorphous, three-dimensional microporous network materials, which have been used in different applications: CO_2_ capture [26], gas separation [27,28], and catalysis [29,30].

Attending to the high Brunauer–Emmett–Teller (BET) surface areas of these POPs which contain 135TPB skeletons [26], and keeping in mind the formation of monophosphine-based POPs by using the knitting technique and its use as efficient catalysts in cross coupling reactions [10,31,32,33,34], we have prepared new materials synthesized by this knitting methodology, using diphosphines instead of monophosphines as palladium anchoring centers and 135TPB and biphenyl (BP) as aromatic skeletons to reduce the phosphine anchoring centers contained in the porous network. The main objective of this work is based on the idea that the presence of diphosphine groups attached by spacers of varying lengths will have important effects on catalysis improvements since the two phosphorus units, when properly arranged in the material, will more efficiently coordinate the metal cation. Also, the stronger phosphorus–metal interaction will reduce leaching, resulting in higher long-term efficiency of these materials as heterogeneous catalysts.

After evaluating the thermal stability and the porous properties of these materials, their catalytic activity has been studied in diverse Suzuki–Miyaura reactions once the palladium(II) acetate has been coordinated into the structure of the microporous material.

## 2. Materials and Methods

### 2.1. Materials

1,3,5-Triphenylbenzene, 99+% (135TPB), biphenyl (BP) and iron(III) chloride anhydrous were obtained from Alfa Aesar; 1,2-bis(diphenylphosphino)ethane (DPPE) was obtained from TCI; 1,3-bis(diphenylphosphino)propane (DPPP) was obtained from Carbosynth; 1,4-bis(diphenylphosphino)butane (DPPB) was obtained from Fluorochem; dimethoxymethane, 99% (DMM) and dichloroethane anhydrous (DCE) were purchased from Sigma-Aldrich. All reagents were used without additional purification.

### 2.2. Techniques

A PerkinElmer Spectrum RX-I FT-IR spectrometer equipped with an ATR accessory Pike GladiATR-210 (GladiATR, Pike Technologies, Fitchburg, WI, USA) was used to record Fourier-transform infrared-attenuated total reflectance (FTIR-ATR) spectra; an Agilent MR-500 MHz (Agilent Technologies, Santa Clara, CA, USA) spectrometer was used to collected ^1^H and ^31^P{^1^H} nuclear magnetic resonance (NMR) spectra. Samples were prepared at 30 mg mL^−1^ using CHCl_3_-d and deuterated trifluoroacetic acid (TFA-d); a Bruker AVANCE 400WB (Bruker, Billerica, MA, USA) spectrometer, equipped with a 89 nm wide bore and a 9.4 T superconducting magnet, operating at a frequency of 100.6 MHz for ^13^C and 162 MHz for ^31^P, using 1 ms contact pulses, a delay time of 3 s, and a rotation rate of 11 kHz was used to record solid state ^13^C and ^31^P cross-polarization magic angle spinning NMR spectra (CP-MAS ^13^C or ^31^P NMR); a TA-Q500 instrument (TA Instruments, New Castle, DE, USA) operating under a continuous nitrogen flow (60 mL min^−1^), with a Hi-Res method at a heating rate of 20 °C min^−1^ and sensitivity and resolution parameters of 1 and 4, respectively, was used to carry out the dynamic thermogravimetric analysis (TGA); a Bruker D8 ADVANCE diffractometer provided with a Goebel Mirror and a pore size distribution (PSD) Vantec detector using Cu Kα (wavelength λ = 1.54 Å) radiation and a step scanning mode with a 2θ step of 0.024°, at a rate of 0.5 s per step, was employed in the reflection mode at room temperature to record wide-angle X-ray scattering (WAXS) patterns; a QUANTA 200 FEG ESEM on Au-metallized samples operating at an acceleration voltage of 1.5 kV in high vacuum and using the method employed for the detection of secondary electrons was used to obtain scanning electron microscopy (SEM) images. The POPs samples were metalized with a ladder of Au (~1 nm) to improve the contrast; the same microscope equipped with EDAX Genesis was used to determine the distribution of Pd in the porous polymer catalysts (Pd@PPs); a SPECS (Germany) device using a PHOIBOS 100 hemispherical electron energy analyzer and a five channel multi-Channeltron detector was used to carry out XPS measurements. The beam source was a nonmonochromatic Mg Kα with a voltage of 12.5 kV and a power of 100 W. The analysis conditions were high-resolution spectra with 10 eV step energy with points acquired every 0.1 eV with a dwell time of 0.5 s. The sample was prepared as follows: the powder sample was placed and stuck on a double-sided carbon sticker and introduced into the XPS sample holder. XPS spectra were not accumulated for a long time (in order to obtain better signal/noise ratios) because Pd tends to change the oxidation state by the effect of the measurement itself. The binding energies were calibrated using the C_1s_ peak at 284.9 eV; Asap 2020 Micromeritics adsorption isotherm equipment was used to measure the N_2_ adsorption−desorption isotherms in the relative pressure range (P/P_0_) going from 10−5 to 1 bar at −196 °C (77 K). Previously, samples were degassed (VacPrep 061 LB Sample Degassing Systems, Micromeritics Instruments. Corp., Norcross, GA, USA) under vacuum at 180 °C for 16 h to remove humidity, adsorbed gases, and solvent from the samples. The adsorption isotherms were used to determine the specific surface area (S_BET_) by applying the Brunauer−Emmett−Teller equation. The pore size was estimated by the Barrett–Joyner–Halenda (BJH) method and the pore volume was determined from the volume of N_2_ adsorbed at 0.98 relative pressure; a radial simultaneous ICP−OES Varian 725-ES device was employed to determine the palladium content in the porous polymer catalysts. An Agilent Technologies 6890N gas chromatograph coupled to Agilent Technologies 5973 inert mass spectrometer was used to determine the yield and conversion of catalytic tests.

The analysis was conducted using a GC-MS system consisting of an Agilent Technologies 6890N gas chromatograph equipped with an HP-1 achiral capillary column (Agilent 19091Z-433, 0.25 mm × 30 m × 0.25 um) and coupled to an Agilent Technologies 5973 inert mass spectrometer. Injection was performed in split mode, starting at 240 °C with a split ratio of 5.78:1 and a split flow rate of 4.2 mL/min. Helium was used as the carrier gas, with a total flow rate of 8.1 mL/min. The initial oven temperature was set to 50 °C, followed by a ramp at a rate of 20 °C/min, reaching a maximum temperature of 300 °C.

The phosphine behavior in acidic media was tested as follows:

Study 1: an NMR tube was charged with 10–12 mg of phosphine (TPP, DPPE, DPPP, or DPPB) and dissolved with 0.6 mL of CDCl_3_/TFA-d (10:1). Then, ^1^H and ^31^P NMR of these solutions were registered.

Study 2: an NMR tube was charged with 0.6 mL of a solution prepared with 1 equivalent of phosphine (65 mg of TPP or 88 mg of DPPE) and 4 equivalents of AlCl_3_ (130 mg (for TPP) or 118 mg (for DPPE)) in 2 mL of CDCl_3_. Then, ^1^H and ^31^P NMR of these solutions were registered.

### 2.3. Synthesis of POPs

**Figure 1:**
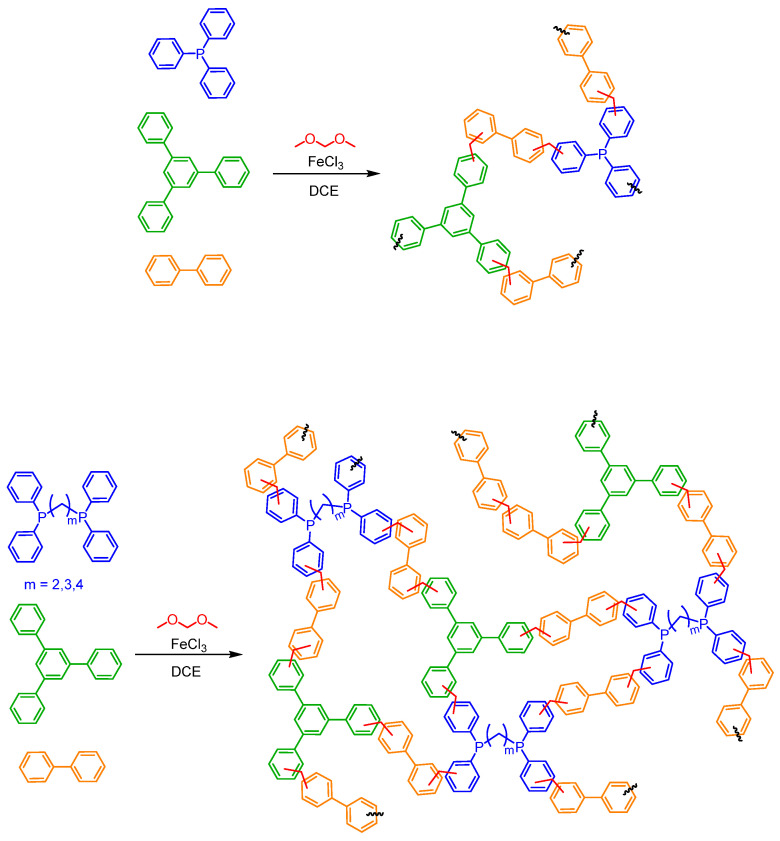
General scheme of phosphine POPs synthetized by knitting.

All porous polymers were obtained following the same synthetic protocol. The acronyms of polymers are shown in Table 1. Also, a general scheme of polymer synthesis is shown in Figure 1.

As an example, the synthesis procedure of PP-3 is described below:

A three-neck Schlenk flask under nitrogen atmosphere was charged with 135TPB (2.36 g, 7.70 mmol) and DPPE (2.30 g, 5.77 mmol). Over a mixture of monomers stirred mechanically at room temperature and under a nitrogen blanket, ferric chloride (14.98 g, 92.37 mmol) was added. The flask was then placed in an ice bath and DCE (97.0 mL) and cold DMM (8.2 mL, 92.36 mmol) were added. The ice bath was maintained for 30 min, and then the mixture was warmed to room temperature and stirred for another 30 min. Subsequently, the mixture was heated to 45 °C and stirred for 24 h. Then it was heated to 80 °C and stirred at this temperature for 72 h. Finally, the reaction was quenched with MeOH, the mixture was filtered, and the obtained polymer was washed at reflux with MeOH in Soxhlet to remove most of the FeCl_3_, with a 4% HCl solution at 60 °C, and with THF at reflux. After drying at 120 °C under a vacuum of 60 mbar for 70 h, the PP-3 polymer was obtained as a brown powder in 79% yield (3.88 g).

### 2.4. Synthesis of Supported Pd(II) Catalysts

All the POPs were charged with Pd(II) in a 1:2 ratio of Pd:diphosphine. The supported Pd(II) catalysts were named Pd@PP-X, where X is the acronym number of the polymer. As an example, the synthesis of Pd@PP-3 is described below:

PP-3 (700.0 mg, 0.8 mmol) was charged in a 20 mL vial and dispersed in 15 mL of dichloromethane (DCM) with an UltraTurrax homogenizer device working at around 10 Krpm. The suspension was switched to a Schlenk flask and rinsed with an additional 15 mL DCM. The mixture was placed under a N_2_ atmosphere and palladium acetate (91.9 mg, 0.4 mmol) was added. The reaction was stirred at room temperature for 48 h in the dark. Then, the polymer network was filtered and washed with DCM and acetone. After drying at 60 °C under a 60 mbar vacuum for 24 h, the supported catalyst was obtained as a dark brown powder (693 mg, 88%).

### 2.5. General Procedure for Suzuki–Miyaura Reactions

Phenyl boronic acid (0.75 mmol), sodium carbonate (1 mmol), and Pd@PP-X catalyst (1 mol%) were added into a 10 mL Schlenk flask. The mixture was blanketed by a nitrogen atmosphere and three consecutive cycles of vacuum/N_2_ process were carried out. Then, the aryl halide derivate (0.5 mmol) and a mixture of ethanol/water (2 mL/3 mL) were added. The reaction vessel was introduced into a preheated aluminum bracket at 80 °C and stirred for 2 h. After the corresponding time, the reaction was cooled down with an ice bath, and the product was extracted with DCM. The product was diluted with acetonitrile, and conversion and yield reaction were obtained by GC-MS by taking all the peaks of the chromatogram as the sum of all reaction products.

### 2.6. Recycling Test

Phenyl boronic acid (0.75 mmol), sodium carbonate (1 mmol), and Pd@PP-3 catalyst (1 mol%) were added into a 10 mL Schlenk flask. The reaction mixture was subjected to three consecutive vacuum/N_2_ cycles under nitrogen atmosphere. Then, 4-bromoanisole (0.5 mmol) and an ethanol/water mixture (2 mL/3 mL) were added. After 30 min of reaction at 80 °C, the solution was cannulated from the reaction vessel keeping the heterogeneous catalyst inside and then the same amount of all reagents and solvents, except the catalyst, were added, repeating the reaction several times. After each cycle, a small sample was taken, and the conversion of the reaction was determined by GC-MS.

### 2.7. Leaching Test

Phenyl boronic acid (0.75 mmol), sodium carbonate (1 mmol), and Pd@PP-3 catalyst (1 mol%) were added into a 10 mL Schlenk flask. The mixture was put under a nitrogen atmosphere by three consecutive cycles of vacuum/N_2_ process, and then 4-bromoanisole (0.5 mmol) and a mixture of ethanol/water (2 mL/3 mL) were added. After 5 min of reaction at 80 °C, the solution was cannulated from the reaction vessel to another vessel through a cotton filter, and then the solution without the POP catalyst was kept for 30 min (two cycles of 15 min) under the same reaction conditions and the progress of the reaction was checked at the end of each cycle. Conversion (%) was obtained by GC-MS.

### 2.8. Scale-Up of Suzuki–Miyaura Reaction

Phenyl boronic acid (15 mmol), sodium carbonate (20 mmol), and Pd@PP-3 catalyst (1 mol%) were added into a 250 mL Schlenk flask. The mixture was blanketed by a nitrogen atmosphere and three vacuum/N_2_ cycles were performed. Then, 4-bromoanisole (10 mmol) and a mixture of ethanol/water (40 mL/60 mL) were added to the Schlenk flask. The reaction vessel was introduced into a preheated aluminum bracket at 80 °C and stirred for 2 h. Afterwards, the reaction was cooled down with an ice bath, and the product was extracted with DCM. The product was diluted with acetonitrile and conversion and the yields were obtained by GC-MS by taking all the peaks of the chromatogram as the sum of all reaction products.

## 3. Results and Discussion

With the idea of knowing the reactivity of these phosphorous nucleophilic monomers and knowing that no TPP-derived homopolymer has been described in the literature, we first tried the reaction using only TPP as an aromatic nucleophilic monomer, employing the classical knitting formation reaction with DMM as the precursor linker, FeCl_3_ as the Lewis acid (molar ratio 1:3:3), and DCE as the solvent. Surprisingly, no polymer network was obtained since the product obtained was soluble in various solvents (acetone and DCM).

This result prompted us to carry out a simple experiment to study the reactivity of the phosphine monomer by ^1^H and ^31^P NMR. Thus, an NMR comparison between phosphine monomers (TPP, DPPE, DPPP, and DPPB) and phosphine monomers treated with a Lewis acid, aluminum chloride (TPP-Al and DPPE-Al), and with a Brönsted acid, TFA-d, (TPP-H, DPPE-H, DPPP-H, and DPPB-H) was carried out. At this point it should be commented that the experimental procedure with aluminum chloride was cumbersome as precipitation occurred in the NMR tube during the NMR measurements, particularly for the DPPP and DPPB phosphines. Therefore, the rest of the experiments with the phosphines DPPP and DPPB were performed only using TFA-d.

**Figure 2:**
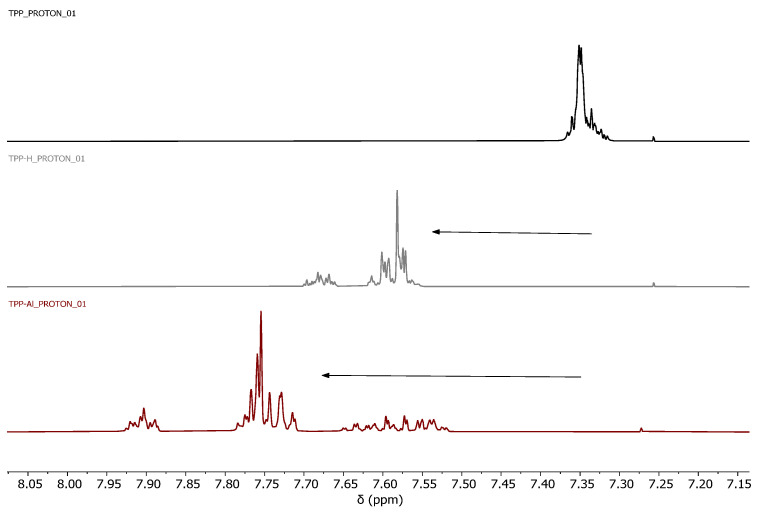
Comparative ^1^H NMR (CDCl_3_). Free TPP (**top**), TPP treated with TFA-d (TPP-H) (**middle**), and TPP treated with AlCl_3_ (TPP-Al) (**bottom**).

**Figure 3:**
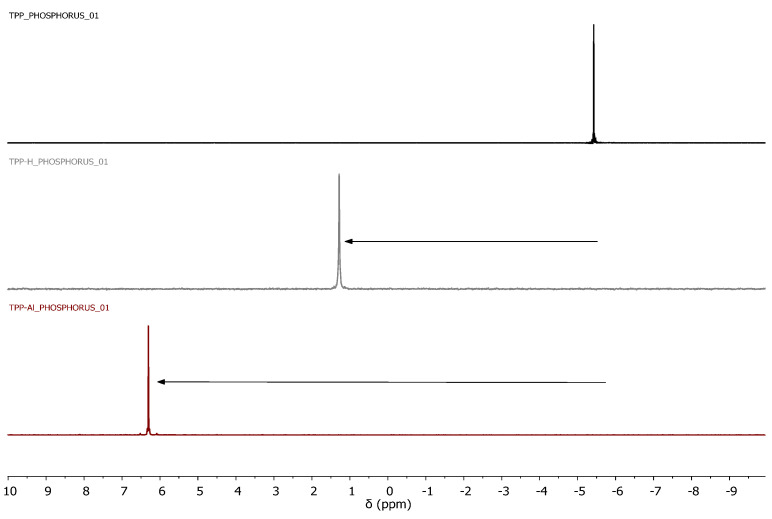
Comparative ^31^P{^1^H}NMR (CDCl_3_). Free TPP (**top**), TPP treated with TFA−d (TPP−H) (**middle**), and TPP treated with AlCl_3_ (TPP−Al) (**bottom**).

It was observed that the phosphorous atom, which is a nucleophile center, could interact with the Lewis acid and also be protonated with the Brönsted acid, which decreased the electronic density of the aromatic rings for both systems. Thus, the signals of all the phosphines after treatment with AlCl_3_ and TFA-d were displaced to a lower field, confirming a reactivity decrease (Figure 2 and Figure 3 and Figures S3–S6 in the Supplementary Materials). Thus, it was clearly observed that the use of either a strong Lewis acid or a Brönsted acid (pKa of TFA is 0.23) brought a clear shift of the aromatic signals of the phosphines at down field. As expected, this signal shift was lower for phosphines treated in TFA-d.

Using this reactivity test, it was shown, together with the mentioned experimental result with TPP, that the phosphorus atom in these reaction conditions, presence of Lewis and Brönsted acids, strongly decreases the reactivity of their aromatic rings, which brings about a lower reactivity. In the next step, the reaction of TPP with 135TPB in the absence or presence of the difunctional comonomer BP (PP-1 and PP-2, respectively) led to the formation of insoluble network copolymers, which could be characterized by FTIR, CPMAS, and WAXS (see Supplementary Materials). A low-intensity ^31^P signal was observed in the ^31^P CPMAS NMR, particularly for the copolymer with BP (PP-2). These results were probably due to the presence of electron-deficient aryl groups in TPP that reduced the reactivity and prevented the whole incorporation of TPP in the final polymer [10].

### 3.1. Synthesis and Characterization of POPs Based on Diphosphines

Considering these results, a new set of POPs based on different diphosphines (DPPE, DPPP, and DPPB) was synthesized by a Friedel–Crafts alkylation reaction. The rigid aromatic building blocks used as nucleophilic monomers (135TPB, diverse diphosphines, and BP as a difunctional monomer) were knitted using DMM as an external crosslinker and FeCl_3_ as Lewis acid catalyst [11]. The polymers were obtained as powders in yields of not less than 75%. The yields of each of these materials are summarized in Table 1. These POPs were insoluble in water, different alcohols, other common solvents such as acetone or DCM, and even in polar aprotic solvents denoting the formation of highly crosslinked polymeric networks.

**Figure 4:**
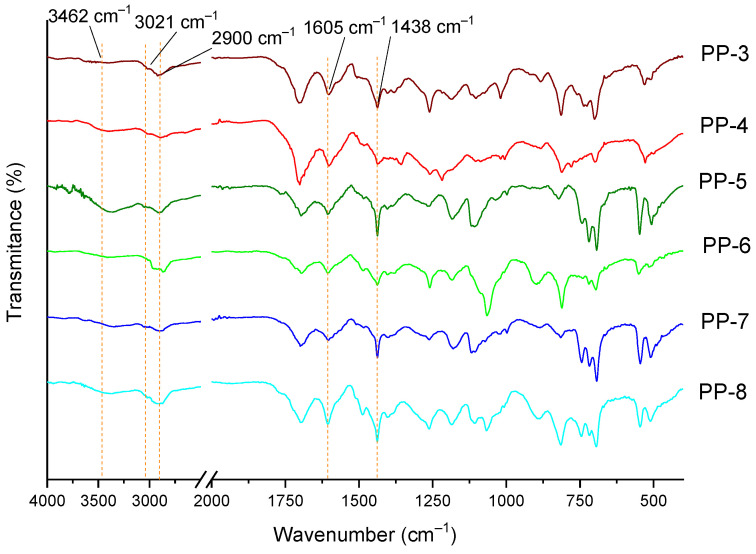
ATR−FTIR spectra of phosphine POPs.

The ATR-FTIR spectrum for all the diphosphine POPs is displayed in Figure 4. The region between 3100 and 3600 cm^−1^ was assigned to humidity traces, peaks around 3020 cm^−1^ were associated with the aromatic C–H bonds in stretching mode, and peaks at 2930 cm^−1^ could be attributed to the stretching alkyl C–H bonds, indicating the existence of methylene linkers formed during the formation of the network, peaks near 1600 cm^−1^ were assigned to C=C aromatic ring skeleton vibrations, whereas a peak at 1440 cm^−1^ was attributed to the CH_2_–C=C bond, which confirms the presence of the linkers between the aromatics moieties [22,35].

The ^13^C CPMAS NMR (Figures S15–S17 Supplementary Materials) showed signals associated with methylene groups at 40 and 20 ppm, corresponding to the cross-linker, and signals from aromatic carbons were observed between 110 and 155 ppm. The ^31^P CPMAS NMR displayed a broad signal centered at 28 ppm, which confirms the presence of diphosphine moieties in the POPs (Figures S19–S21 Supplementary Materials).

**Figure 5:**
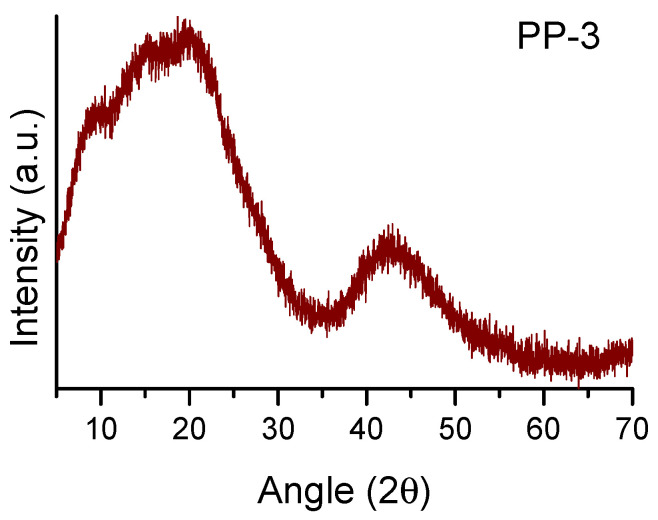
WAXS patterns of PP-3.

The nature of these POPs was confirmed by WAXS, where an amorphous halo was observed. Figure 5 shows the WAXS pattern of PP-3 as an example (the WAXS graphs of the other PPs are displayed in the Supplementary Materials Figures S23–S28). The amorphous halo showed at least four maxima at 9.5, 15.5, 19.5, and 42.5°, which indicates the presence of some regularity in the chain packing, as was previously observed for other polymers synthesized from 135TPB [26,29]. These four maxima correspond to 1.0, 0.6, 0.4, and 0.2 nm intersegmental distances, respectively.

The thermal stability of these POPs was determined by dynamic TGA registered under a nitrogen atmosphere in the temperature range of 30 to 800 °C. All the POPs showed a small weight loss (less than 6%) under 200 °C due to humidity, solvent, and adsorbed gases. It is noteworthy that the thermal stability of POP copolymers with BP was higher than those POPs without BP. The char yield of POPs was higher than 70% at 800 °C, except for PP-5 and PP-7, in polymers synthesized without BP. The thermograms of these POPs are displayed in Figures S29–S30 in Supplementary Materials.

The morphology of POPs studied by SEM showed the presence of spherical particles with diameters between 70 and 150 nm (Figures S41–S46 in the Supplementary Materials).

**Figure 6:**
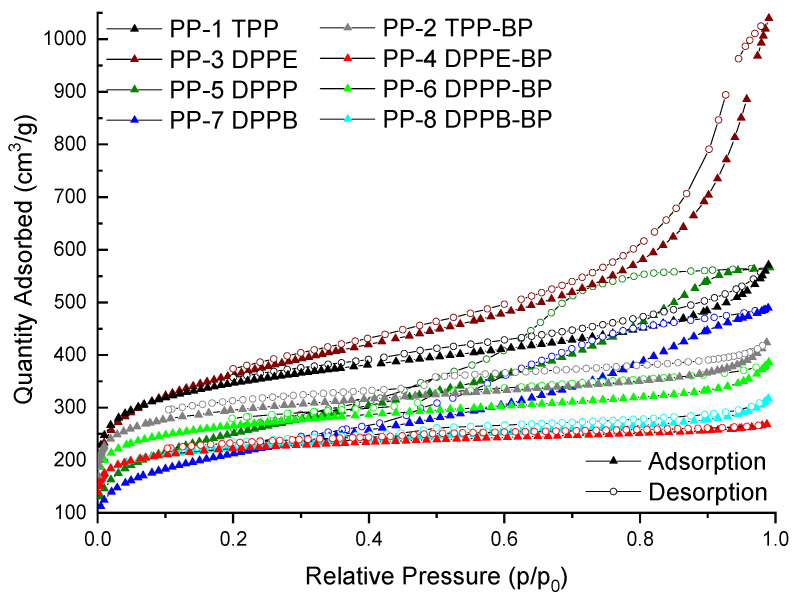
Nitrogen adsorption (solid symbol) and desorption (open symbol) isotherms of PP supports.

The porosity features of these POPs were studied from the N_2_ adsorption/desorption isotherms at 77 K. These materials showed a large surface area, in the range 760–1300 m^2^/g (see Table S1, Supplementary Materials). The adsorption–desorption isotherm curves are displayed in Figure 6. All the PPs materials showed a very significant N_2_ uptake at low relative pressures (P/P_0_ < 0.01) in the adsorption branch and hysteresis cycles, which denoted the presence of significant microporosity [36,37]. The PPs synthesized from BP (PP-4, PP-6, and PP-8), showed isotherms similar to IUPAC Type I, indicating that these materials showed a mainly microporous character, while PP-3, PP-5, and PP-7 showed isotherms Type I + Type IV, indicating the existence of both microporosity and mesoporosity. Attending to the porosity contribution (Table S1 in Supplementary Materials), the PPs synthesized with BP showed a microporosity higher than 50%, while PPs synthesized without BP showed a higher contribution of meso- and macropores in their structures. These results show that the presence of BP in these polymeric networks results in more microporous materials.

### 3.2. Synthesis and Characterization of Pd@PP-X Catalysts

The diphosphine polymers were coordinated to palladium(II) acetate by treatment with a palladium acetate solution in DCM. The Pd@PP-X catalysts were characterized by FTIR, CPMAS (^13^C and ^31^P), and WAXS. The palladium content in the catalysts was studied by ICP-OES (Table 2). It should be noted that the longer the aliphatic chain of diphosphine the more palladium the polymer networks incorporated.

The ATR-FTIR of Pd catalysts (Figures S8–S13 in Supplementary Materials) showed absorption bands around 1700 and 1600 cm^−1^ and also at 1100–1300 cm^−1^, which could be attributed to the C=O tension of palladium acetate. The CPMAS NMR peaks of these catalysts are displayed in the Supplementary Materials Section (Figures S15–S21). Both ^13^C and ^31^P show hardly any differences with respect to the corresponding spectra of the derivatives without Pd, a fact that had already been observed in polymers based on triphenylphosphine [22].

The WAXS spectra of the POP-supported catalysts showed significant changes when compared to the WAXS patterns of the precursor POPs (Figures S23–S28 in Supplementary Materials). These changes were especially noticeable in the case of Pd@PP-3 and Pd@PP-6. Pd@PPs showed very weak reflection peaks around 12 and 33° (peaks that can be associated with Pd(OAc)_2_ units) [36].

The BET surface areas and porosity of the catalysts were studied by adsorption–desorption N_2_ isotherms at 77 K. For the materials loaded with palladium acetate, small changes in porosity were observed mainly in the isotherms of the polymer networks synthesized using BP, whereas a much more significant change was observed in the mesoporosity of the materials synthesized with or without BP (Figures S32–S40 in Supplementary Materials). Finally, changes in the porosity distribution in the mesopore zone were observed for some of the mesoporous materials.

**Figure 7:**
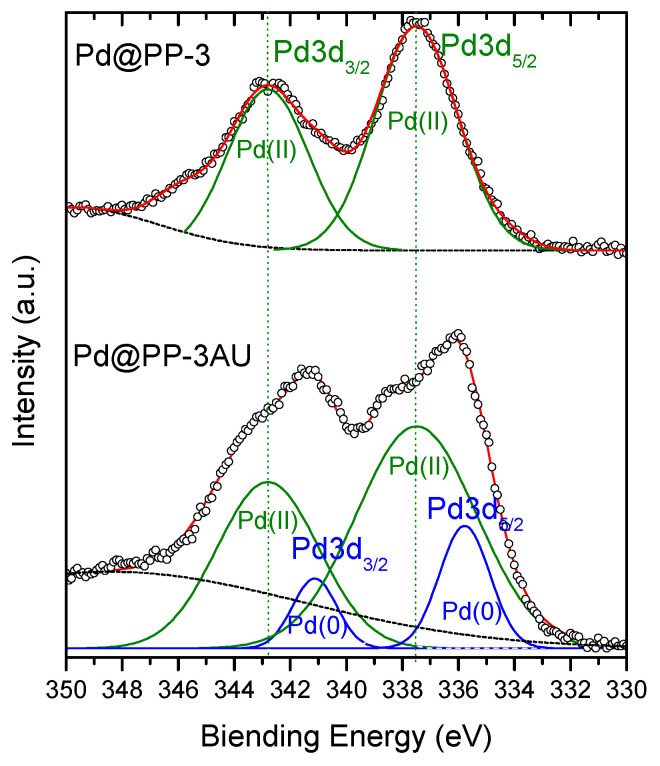
XPS analysis of Pd3d binding energies for Pd@PP3 before (**top**) and after 2 catalytic cycles of two hours (Pd@PP-3AU) (**bottom**).

X-ray photoelectron spectroscopy (XPS) analysis was performed for Pd@PP-3 before and after use for 2 catalytic cycles of 2 h (so-called Pd@PP-3AU) to investigate the oxidation states of palladium species in these materials (Figure 7). The Pd 3d spectra presented two sets of peaks corresponding to Pd 3d_5/2_ and Pd 3d_3/2_. The Pd 3d_5/2_ and Pd 3d_3/2_ binding energy peaks in Pd@PP-3 located at 337.5 and 342.8 eV, respectively, were assigned to Pd(II) species, whereas the Pd 3d_5/2_ and Pd 3d_3/2_ peaks located at 336.1 and 341.4 eV in Pd@PP-3AU were associated with Pd(0) species.

### 3.3. Catalytic Activity

The catalytic activity of these heterogeneous catalysts was evaluated by carrying out Suzuki–Miyaura cross-coupling reactions using similar conditions to previous catalytic experiments performed by our research group [29]. Thus, the conditions used in the optimization studies were 1 eq of aryl halide, 1.5 eq of boronic acid, 2 eq of sodium carbonate, 1 mol% of catalyst with respect to the aryl halide, a mixture of ethanol/water (2:3) as the solvent, and maintaining the reaction for 2 h at 80 °C (conversion was estimated by GC-MS). Table 3 shows the catalytic study results. A control test was performed with the POPs without palladium acetate, observing no catalytic activity.

As a first approximation of the catalytic activity of Pd@PPs, the reaction with 4-bromoanisole and phenylboronic acid, under the conditions described above, gave, for all reactions, excellent conversions to the desired coupling product (Table 3, entries 1–6). Additional studies were carried out using different aryl bromides (Table 3, entries 7–24). Little amounts of homocoupling products were detected under these conditions due to the use of an excess of the boronic acid that is necessary to increase the rate of the transmetallation reaction.

Among all the Pd@PPs, Pd@PP-3 was chosen, as dppe was the most affordable diphosphine. Then, additional studies (Table 4) were carried out using this catalyst with different aryl halides. Among them, the experiments with aryl chlorides gave high yields for the coupling products reinforcing the importance of our materials as catalysts in these reactions. However, when a bulky aryl chloride was used (i.e., 1-chloronaphthalene) a lower reactivity was achieved (22% yield, 30% conversion).

**Figure 8:**
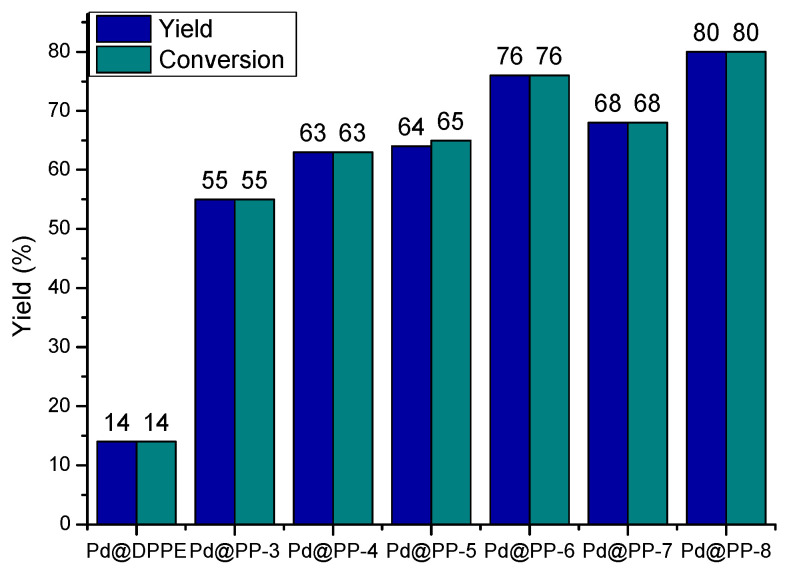
Comparative study between Pd-PP under milder reaction conditions: 15 min., 40 °C and 0.1 mol% of Pd-PP.

Considering the exceptional performance of these catalytic materials with bromide derivatives, we embarked on further investigations aimed at optimizing their catalytic properties. Specifically, we decreased the reaction temperature to 40 °C, reduced the reaction time to 15 min, and minimized the catalyst loading from 1 to 0.1 mol% to evaluate their ultimate catalytic potential (Figure 8). Additionally, we conducted a comparative analysis, pitting the results obtained with a polymeric network-derived catalyst against those of a model homogeneous catalyst (Pd@DPPE, as detailed in Supplementary Materials Section S1), in order to discern the distinctions between homogeneous and heterogeneous catalysts.

From these catalytic results, turnover number (TON) and turnover frequency (TOF) values at 40 °C were determined, to define the catalytic capacity of these new materials. These values are presented in Table 5. The best values of TON and TOF were observed for the polymer derived from DPPB, the phosphine with the longest chain between phosphorous atoms, (Pd@PP-8). For all materials, the higher TON and TOF values were found for the polymer materials derived from BP.

### 3.4. Recyclability of Pd@PPs

**Figure 9:**
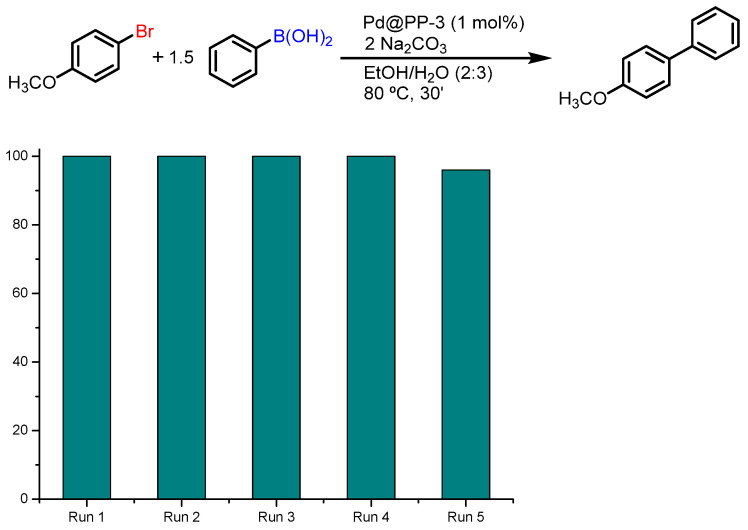
Recyclability of Pd@PP-3.

To demonstrate the catalytic activity of these materials in industrial processes, a recyclability study was performed using 4-bromoanisole as the aryl derivative and Pd@PP-3 (1 mol%) as the catalyst. As shown in Figure 9, no appreciable change in reactivity was observed by repeating the same procedure for at least 5 runs, confirming that the palladium moiety is anchored to the active site and no substantial decrease in reactivity was observed for these materials after several uses.

### 3.5. Leaching Test

To demonstrate that the palladium is strongly anchored to the active site of the catalytic support, a leaching test was performed studying the coupling reaction between 4-bromoanisol and phenyl boronic acid at 80 °C using Pd@PP-3 (1 mol%) as the catalyst in a short time (5 min) to avoid the complete conversion. Afterwards, the 5 min reaction was cannulated to another vessel, and the filtered solution was immediately checked by GC-MS. This process was also monitored after 20 min and 35 min. Once the catalyst was removed, no increment in the conversion was observed, confirming that the palladium acetate was firmly anchored in the support (Table S2 and Figure S50 in the Supplementary Materials).

### 3.6. Scale-Up Catalyzed Suzuki–Miyaura Reaction Procedure

A scale-up experiment of Suzuki–Miyaura reaction was accomplished to show the possibility of using these polymers in large reaction batches. The proof of concept was made using 4-bromoanisole as the bromoaryl derivate and Pd@PP-3 as the catalyst, using the same conditions depicted in Table 4. The final conversion was almost quantitative (98%).

## 4. Conclusions

The positive effects of these Pd(II)-loaded diphosphine-based microporous polymer networks on their efficiency as catalysts and on the reduction of leaching processes have been evident, and it could be stated that these materials compete in Suzuki–Miyaura reactions with TPP-derived microporous networks, with the added advantage of their greater long-term stability.

The primary goal of this research is rooted in the concept that incorporating diphosphine groups will significantly enhance catalysis. The arrangement of these two phosphorus units within the material is expected to facilitate more efficient complexation with the metal cation. Additionally, the strengthened P–metal interaction is anticipated to minimize metal loss to a greater extent, leading to increased long-term efficiency.

In this research work, a set of new amorphous microporous polymeric networks, POPs, derived from diphosphines, and capable of anchoring Pd(II) in their porous structures, have been obtained. These POPs were synthesized by using a knitting technology from a trifunctional aromatic monomer, 135TPB, or from a mixture of 135TPB and the difunctional monomer BP, and different diphosphines (DPPE, DPPP, or DPPB), employing FeCl_3_ as a promoter catalyst and DMM as a precursor linker. These polymeric networks were microporous and showed high BET surface areas (above 760 m^2^/g) as well as moderate thermal stability. Copolymers synthesized using BP exhibited higher microporosity, while those synthesized without BP, although microporous, showed a higher contribution of mesopores. These palladium-anchored POPs gave excellent results as catalysts in Suzuki–Miyaura reactions, showing excellent TON and TOF values. The excellent catalytic activity was maintained for at least five consecutive cycles. It was observed that polymeric networks formed from diphosphines possessing the longest aliphatic chain (DPPB-derived POPs) showed the best catalytic activity. Also, the copolymer networks, derived from BP, showed better catalysis results than the polymeric networks not derived from BP. Finally, a leaching test confirmed that the loss of Pd(II) acetate was greatly minimized, indicating that the palladium cation was well anchored in the phosphine units of these heterogeneous catalytic materials.

## Figures and Tables

**Table 1:** Polymer acronyms and reaction yields.

Polymers (Acronyms)		R (%)
PP-1	135TPB-TPP-DMM (1:1:3)	75
PP-2	135TPB-TPP-BP-DMM (1:1:3:6)	99
PP-3	135TPB-DPPE-DMM (4:3:12)	79
PP-4	135TPB-DPPE-BP-DMM (4:3:12:24)	92
PP-5	135TPB-DPPP-DMM (4:3:12)	90
PP-6	135TPB-DPPP-BP-DMM (4:3:12:24)	99
PP-7	135TPB-DPPB-DMM (4:3:12)	86
PP-8	135TPB-DPPB-BP-DMM (4:3:12:24)	100

The polymers were synthesized at 45 °C for 24 h and 80 °C for 72 h.

**Table 2:** Palladium(II) content (%) in the Pd@PP-X catalysts.

Pd@PP	%Pd Content ICP-OES
Pd@PP-3	4.96
Pd@PP-4	1.06 *
Pd@PP-5	5.08
Pd@PP-6	1.71 *
Pd@PP-7	5.86
Pd@PP-8	2.83

* The low Pd values probably reflect a lower phosphorous content in the corresponding polymers.

**Table 3:** Suzuki−Miyaura reactions between different aryl bromides and phenylboronic acid catalyzed by the Pd@PP-X catalysts.

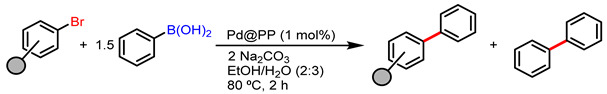				
Entry	Ar-Br	Pd-PP	Conversion (%)	Relative Yield Hetero: Homocoupling (%)
1	4-Bromoanisole	Pd@PP-3	100	100:0
2		Pd@PP-4	100	100.0
3		Pd@PP-5	100	100:0
4		Pd@PP-6	100	100:0
5		Pd@PP-7	100	100:0
6		Pd@PP-8	100	100:0
7	4-Bromobiphenyl	Pd@PP-3	94	87:8
8		Pd@PP-4	100	98:2
9		Pd@PP-5	100	98:2
10		Pd@PP-6	100	98:2
11		Pd@PP-7	100	97:3
12		Pd@PP-8	100	98:2
13	1-Bromonaphtalene	Pd@PP-3	97	96:1
14		Pd@PP-4	100	99:0
15		Pd@PP-5	100	99:0
16		Pd@PP-6	100	99:0
17		Pd@PP-7	100	99:0
18		Pd@PP-8	100	98:0
19	9-Bromophenanthrene	Pd@PP-3	100	96:2
20		Pd@PP-4	100	100:0
21		Pd@PP-5	100	100:0
22		Pd@PP-6	100	100:0
23		Pd@PP-7	97	94:3
24		Pd@PP-8	98	98:0

**Table 4:** Suzuki−Miyaura reactions of aryl bromides and chlorides with phenylboronic acid catalyzed by the Pd@ PP-3 catalyst.

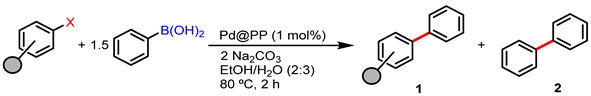				
Entry	Aryl Halide	Pd-PP	Conversion (%)	Relative Yield Hetero: Homocoupling (%)
25 ^a^	4-Bromoanisole	PP-3	0	0:0
26 ^b^	4-Bromoanisole	Pd@PP-3	90	82:8
27	Bromobenzene ^c^	Pd@PP-3	100	100:0
28	4-Bromotoluene	Pd@PP-3	100	100:0
29	1-Bromo-4-(tert-butyl)benzene	Pd@PP-3	100	98:1
30	Bromopentamethylbenzene	Pd@PP-3	100	15:3
31	2-Bromo-1,3,5-triisopropilbenceno	Pd@PP-3	43	10:3
32	4-Chloroanisole	Pd@PP-3	100	85:0
33	Chlorobenzene ^c^	Pd@PP-3	100	92:0
34	4-Chlorotoluene	Pd@PP-3	81	59:12
35	1-Chloronaphthalene	Pd@PP-3	30	22:6

^a^ Study accomplished using the POP non-loaded with palladium. ^b^ Study accomplished using only water as solvent. ^c^ Yield refers to the coupling product, which, in these cases, cannot distinguish between species **1** and **2** (i.e., between hetero- or homocoupling products).

**Table 5:** TON and TOF values of the diphosphine POPs catalysts.

	TON	TOF (h^−1^)
Pd@PP-3	548	2192
Pd@PP-4	625	2500
Pd@PP-5	647	2588
Pd@PP-6	762	3048
Pd@PP-7	677	2708
Pd@PP-8	802	3208

## Data Availability

Not applicable.

## Supplementary Materials

The following supporting information can be downloaded at: https://www.mdpi.com/article/10.3390/polym15204143/s1, Figure S1: ^1^H-NMR (CD_2_Cl_2_) of DPPE-Pd(II) catalyst; Figure S2: ^31^P-NMR (CD_2_Cl_2_) of DPPE-Pd(II) catalyst; Figure S3: Comparative ^1^H NMR (CDCl_3_) between phosphines without acid treatment (TPP, dppe, dppp, and dppb) and phosphines treated with TFA-d (TPP-H, dppe-H, dppp-H, and dppb-H); Figure S4: Comparative ^31^P NMR (CDCl_3_) between phosphines without acid treatment (TPP, dppe, dppp, and dppb) and phosphines treated with TFA-d (TPP-H, dppe-H, dppp-H, and dppb-H); Figure S5: Comparative ^1^H NMR (CDCl_3_) between DPPE without and with treatment with TFA-d (dppe-H) and with AlCl_3_ (dppe-Al); Figure S6: Comparative ^31^P NMR (CDCl_3_) between DPPE without and with treatment with TFA-d (dppe-H) and with AlCl3 (dppe-Al); Figure S7. ATR-FTIR of PP-1 and PP-2; Figure S8: ATR-FTIR of Pd@PP-3 and its PP-3 precursor; Figure S9: ATR-FTIR of Pd@PP-4 and its PP-4 precursor; Figure S10: ATR-FTIR of Pd@PP-5 and its PP-5 precursor; Figure S11: ATR-FTIR of Pd@PP-6 and its PP-6 precursor; Figure S12: ATR-FTIR of Pd@PP-7 and its PP-7 precursor; Figure S13: ATR-FTIR of Pd@PP-8 and its PP-8 precursor; Figure S14: CP/MAS ^13^C NMR spectra of PP-1 and PP-2 obtained from TPP; Figure S15: CP/MAS ^13^C NMR spectra of PP supports and Pd catalysts obtained from DPPE; Figure S16: CP/MAS ^13^C NMR spectra of PP supports and Pd catalysts obtained from DPPP; Figure S17: CP/MAS ^13^C NMR spectra of PP supports and Pd catalysts obtained from DPPB; Figure S18: CP/MAS ^31^P NMR spectra of PP-1 and PP-2 obtained from TPP; Figure S19: CP/MAS ^31^P NMR spectra of PP supports and Pd catalysts obtained from DPPE; Figure S20: CP/MAS ^31^P NMR spectra of PP supports and Pd catalysts obtained from DPPP; Figure S21: CP/MAS ^31^P NMR spectra of PP supports and Pd catalysts obtained from DPPB; Figure S22: WAXS pattern of PP-1 and PP-2; Figure S23: WAXS pattern of Pd@PP-3 and its PP-3 precursor; Figure S24: WAXS pattern of Pd@PP-4 and its PP-4 precursor; Figure S25: WAXS pattern of Pd@PP-5 and its PP-5 precursor; Figure S26: WAXS pattern of Pd@PP-6 and its PP-6 precursor; Figure S27: WAXS pattern of Pd@PP-7 and its PP-7 precursor; Figure S28: WAXS pattern of Pd@PP-8 and its PP-8 precursor; Figure S29: Thermograms of phosphine POPs without BP; Figure S30: Thermograms of phosphine POPs with BP; Figure S31: Thermograms of phosphine POPs-Pd(II) catalysts; Figure S32: N_2_ adsorption (full symbols)/desorption (empty symbols) isotherms of polymers synthetized from DPPE measured at 77 K; Figure S33: N_2_ adsorption (full symbols)/desorption (empty symbols) isotherms of polymers synthetized with DPPP measured at 77 K; Figure S34: N_2_ adsorption (full symbols)/desorption (empty symbols) isotherms of polymers synthetized with DPPB measured at 77 K; Figure S35: Adsorption porous distribution of network polymers obtained from DPPE; Figure S36: Adsorption porous distribution of network polymers obtained from DPPP; Figure S37: Adsorption porous distribution of network polymers obtained from DPPB; Figure S38: Desorption porous distribution of network polymers obtained from DPPE; Figure S39: Desorption porous distribution of network polymers obtained from DPPP; Figure S40: Desorption porous distribution of network polymers obtained from DPPB; Figure S41: SEM image of PP-3; Figure S42: SEM image of PP-4; Figure S43: SEM image of PP-5; Figure S44: SEM image of PP-6; Figure S45: SEM image of PP-7; Figure S46: SEM image of PP-8; Figure S47: SEM-EDX of Pd@PP-3; Figure S48: XPS analysis of Pd3d binding energies for Pd@PP3; Figure S49: XPS analysis of Pd3d binding energies for Pd@PP4; Figure S50: Leaching test of Pd@PP-3. Table S1: Porosity Parameters derived from N_2_ adsorption isotherms; Table S2: Leaching test.
