# Supplementary material for: Palladium Catalysts Supported in Microporous Phosphine Polymer Networks
Source: Polymers (Basel). 2023 Oct 19;15(20):4143. doi: 10.3390/polym15204143 (PMC10611190; doi:10.3390/polym15204143)
Supplement: Supplementary file 1 [file polymers-15-04143-s001.zip › polymers-2646968-supplementary.pdf]

polymers

Supplementary Materials

Palladium Catalysts Supported in Microporous Phosphine Polymer Networks

Noelia Esteban [1], Miguel Claros [1], Cristina Álvarez [1,2,3], Ángel E. Lozano [1,2,3], Camino Bartolomé [1], Jesús M. Martínez-Ilarduya [1] and Jesús A. Miguel [1]

[1] IU CINQUIMA, School of Sciences, University of Valladolid, Paseo Belén 5, E-47011 Valladolid, Spain
[2] SMAP, UA-UVA_CSIC, Associated Research Unit to CSIC. University of Valladolid, School of Sciences, Paseo Belén 7, E-47011 Valladolid, Spain
[3] Institute of Polymer Science and Technology, ICTP-CSIC, Juan de la Cierva 3, E- 28006 Madrid, Spain
* Correspondence: caminob@qi.uva.es (C.B) and jamiguel@uva.es (J.A.M.)

CONTENTS

Section 1. Synthesis and characterization of homogenous DPPE-Pd(II) catalysts

Section 2. NMR reactivity study of phosphines

Section 3. Characterization of phosphine POPs and Pd(II)-supported catalysts

Section 3.1. ATR-FTIR of polymer networks

Section 3.2. CPMAS Solid State NMR of polymer networks

Section 3.3. WAXS of polymer networks

Section 3.4. Dynamic thermogravimetric analysis (TGA) of polymer networks

Section 3.5. Gas adsorption isotherms and pore distribution of polymer networks

Section 3.6. SEM and SEM-EDX characterization of Pd-supported catalysts

Section 3.7. XPS of Pd catalysts

Section 4. Catalytic activity

Section 1. Synthesis and characterization of homogenous DPPE-Pd(II) catalyst

A homogenous catalyst (Pd@DPPE) was synthesized in order to compare with the PPs. Pd@DPPE was synthetized as follows: A Schlenk flask was charged with DPPE (600.0 mg) and palladium acetate (338.1 mg). Three cycles of vacuum/N_2 were realized and then 20 mL of dry DCM were added. The mixture was stirred at room temperature in the dark for 15 min. Then, the volume of the reaction was halved by evaporating the solvent under vacuum, and 20 mL of diethyl ether was added. The solid was filtered and washed with two fractions of 10 mL of cold diethyl ether. The catalyst was obtained as a yellow powder (705.6 mg, 75.2%).

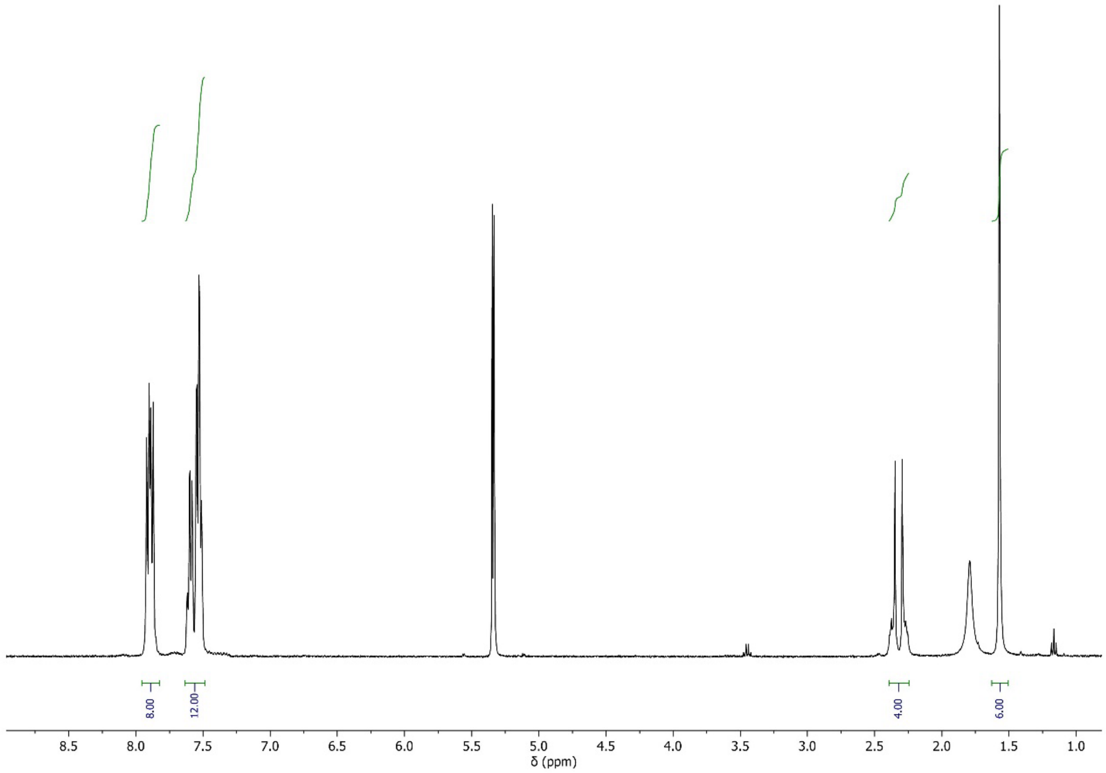

Figure S1. ^{1}H-NMR (CD_2Cl_2) of DPPE-Pd(II) catalyst.

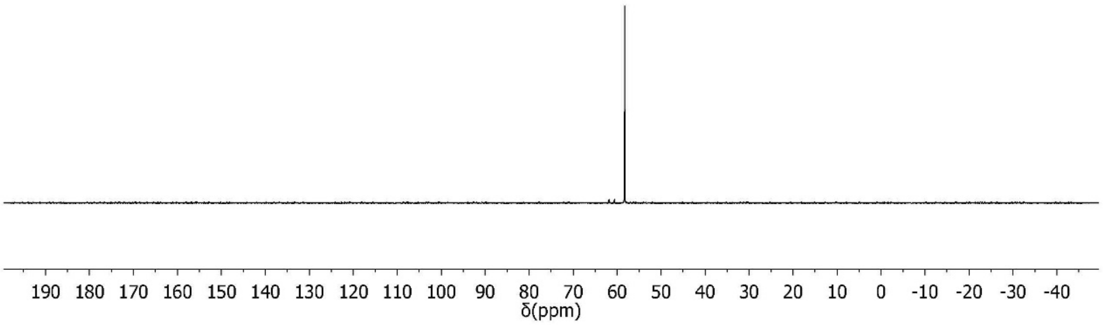

Figure S2. ^{31}P-NMR (CD_2Cl_2) of DPPE-Pd(II) catalyst.

Section 2. NMR reactivity study of phosphines

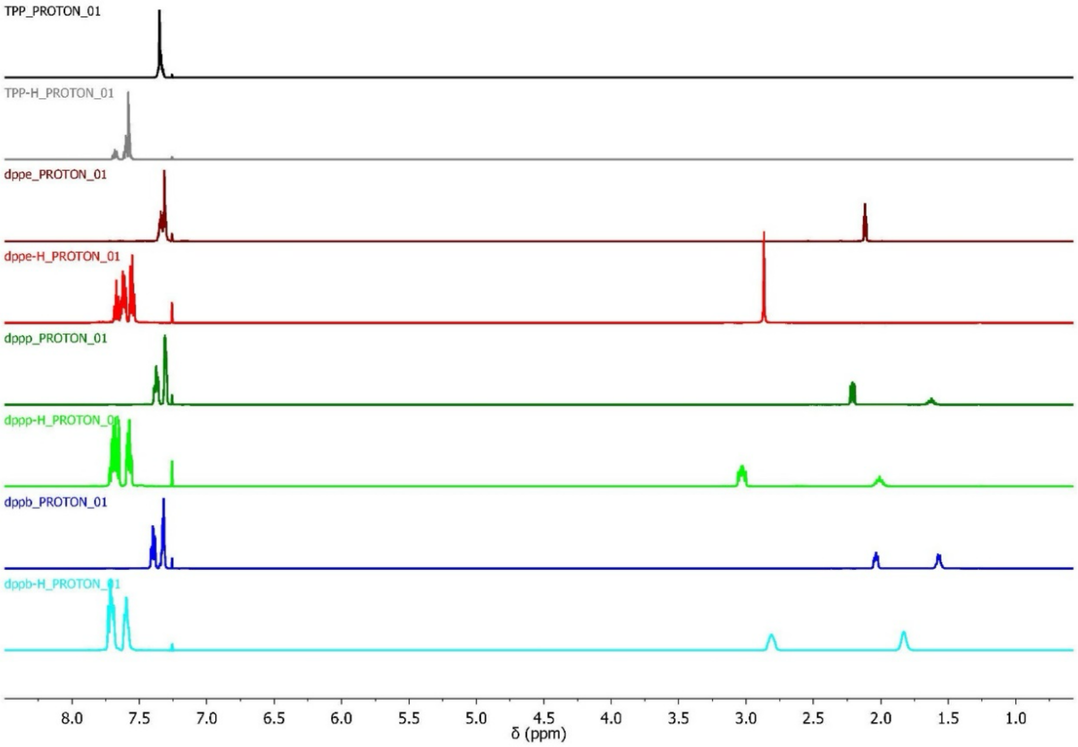

Figure S3. Comparative ^{1}H NMR ($CDCl_3$) between phosphines without acid treatment (TPP, dppe, dppp, dppb) and phosphines treated with TFA-d (TPP-H, dppe-H, dppp-H, dppb-H).

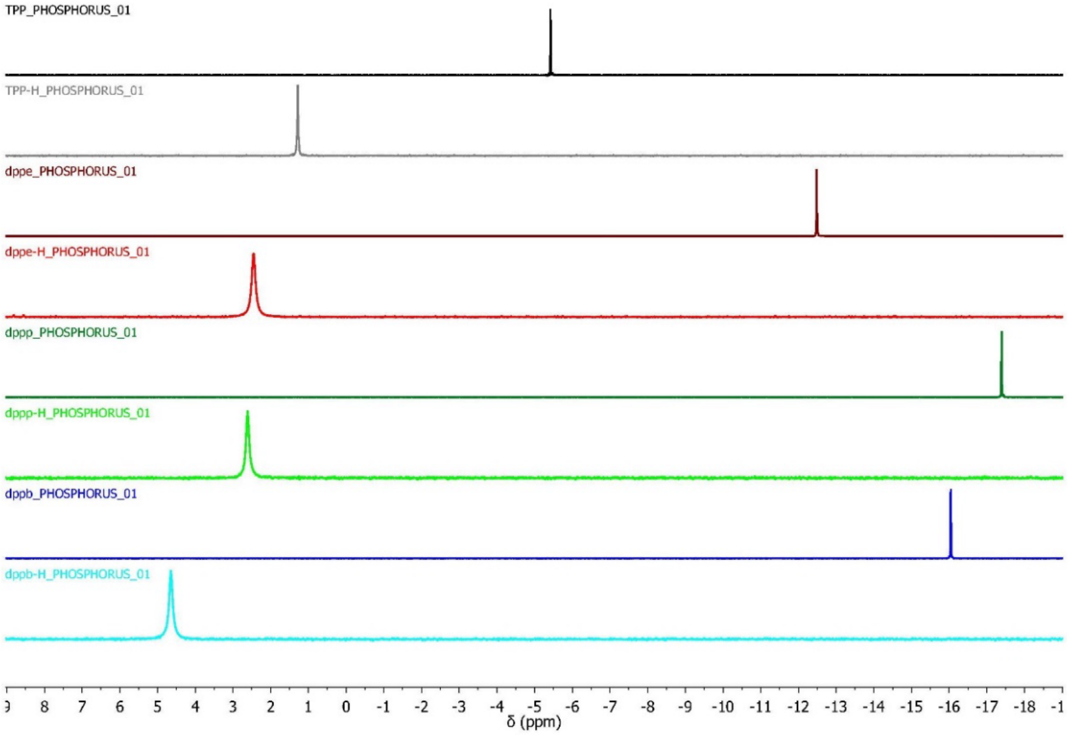

Figure S4. Comparative ^{31}P NMR ($CDCl_3$) between phosphines without acid treatment (TPP, dppe, dppp, dppb) and phosphines treated with TFA-d (TPP-H, dppe-H, dppp-H, dppb-H).

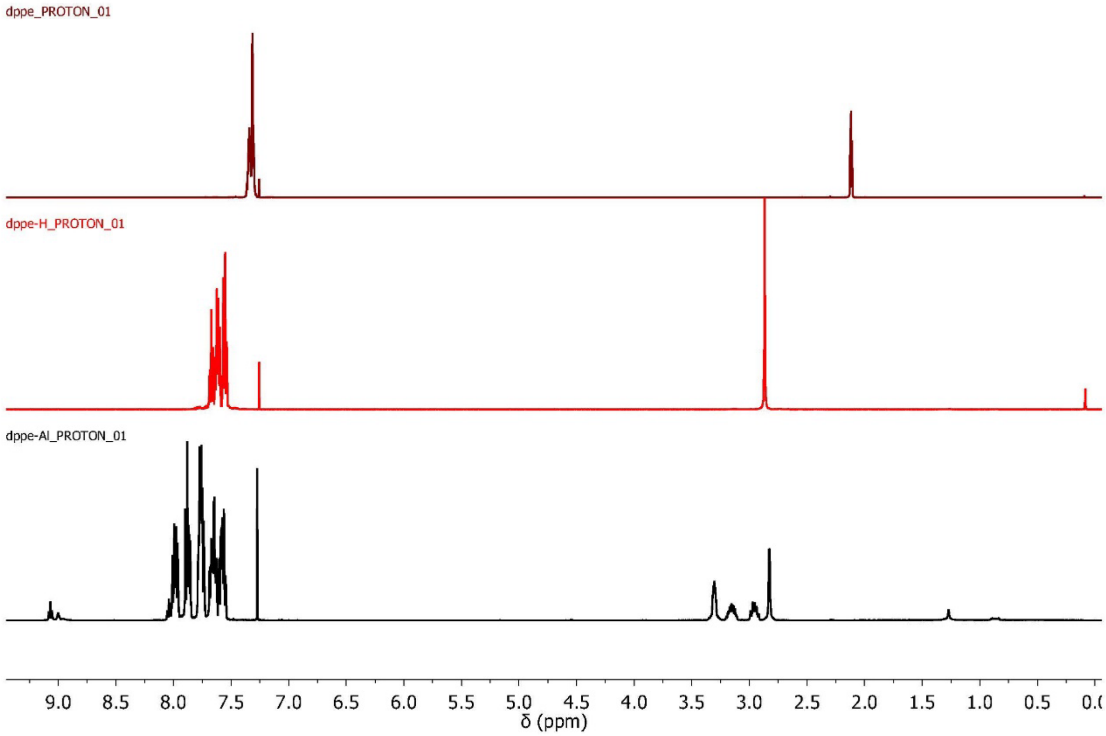

Figure S5. Comparative ^{1}H NMR ($CDCl_3$) between DPPE without and with treatment with TFA-d (dppe-H) and with $AlCl_3$ (dppe-Al).

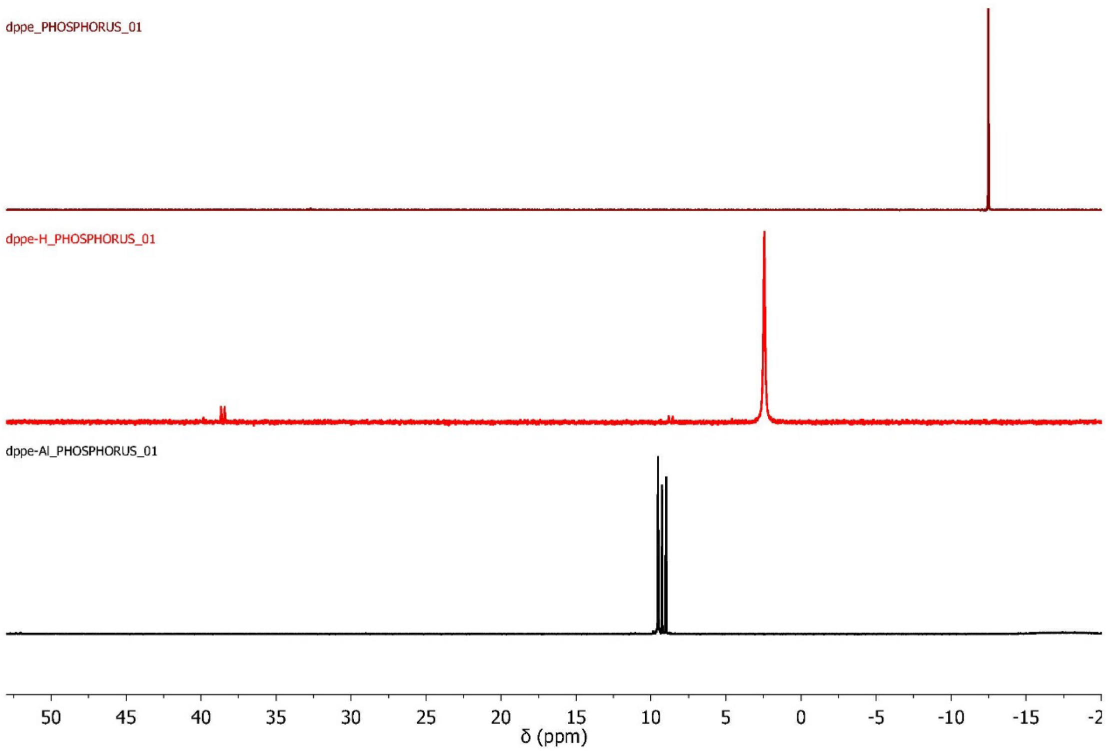

Figure S6. Comparative ^{31}P NMR ($CDCl_3$) between DPPE without and with treatment with TFA-d (dppe-H) and with $AlCl_3$ (dppe-Al).

Section 3. Characterization of phosphine POPs and Pd(II)-supported catalysts

Section 3.1. ATR-FTIR of polymer networks

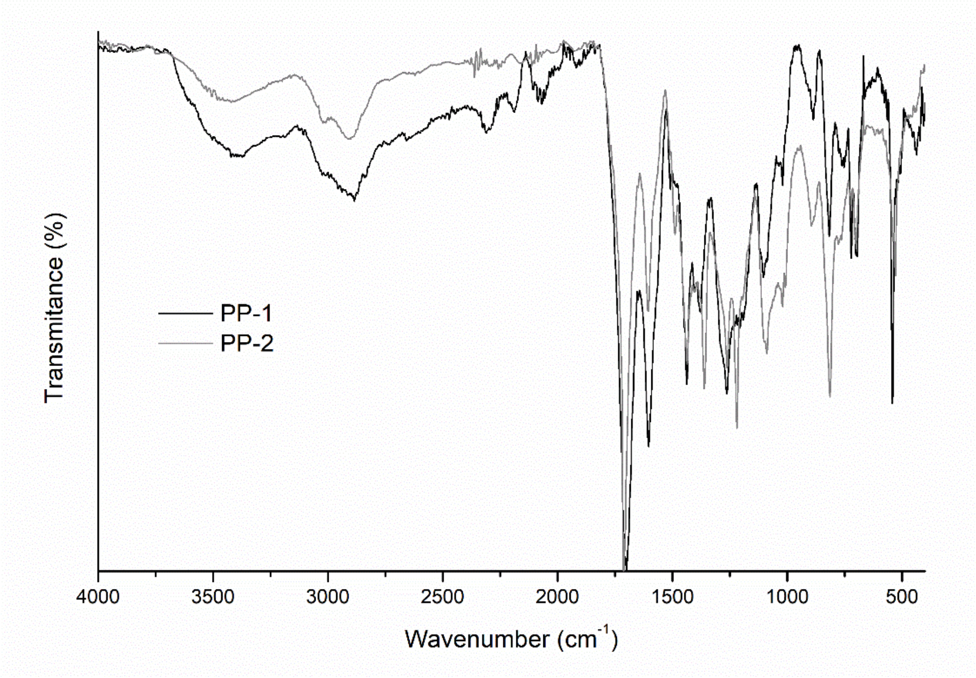

Figure S7. ATR-FTIR of PP-1 and PP-2.

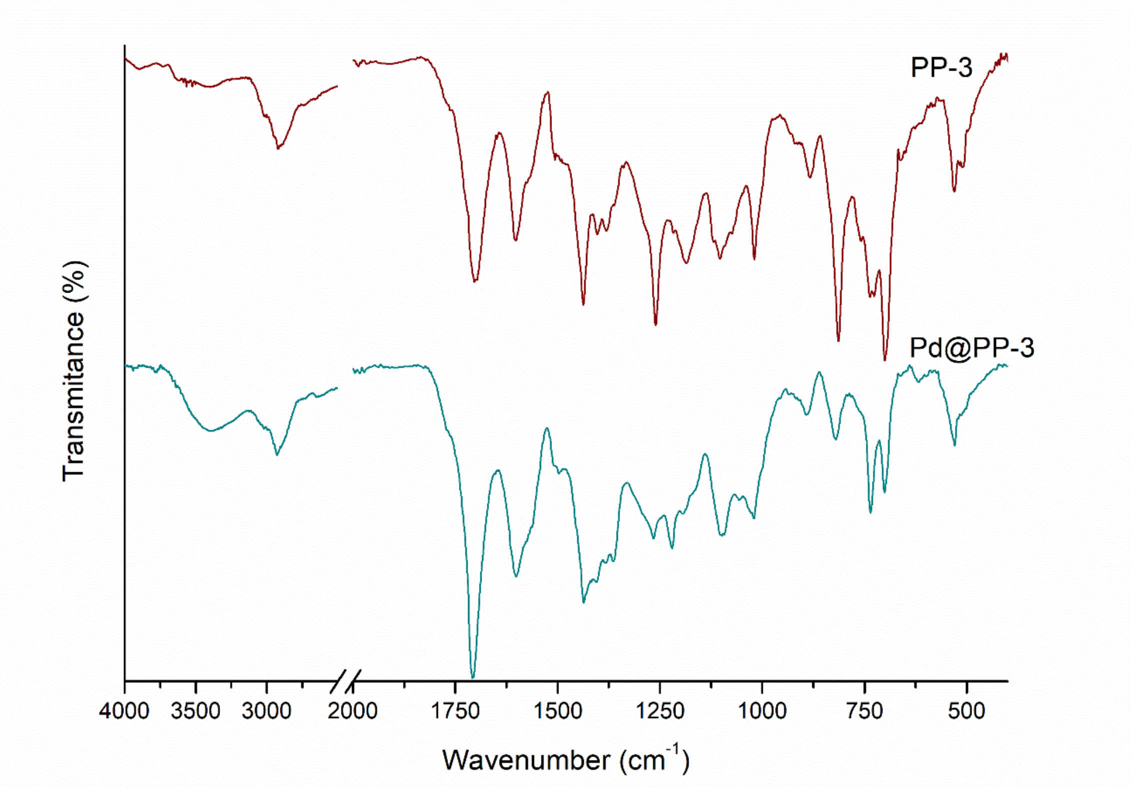

Figure S8. ATR-FTIR of Pd@PP-3 and its PP-3 precursor.

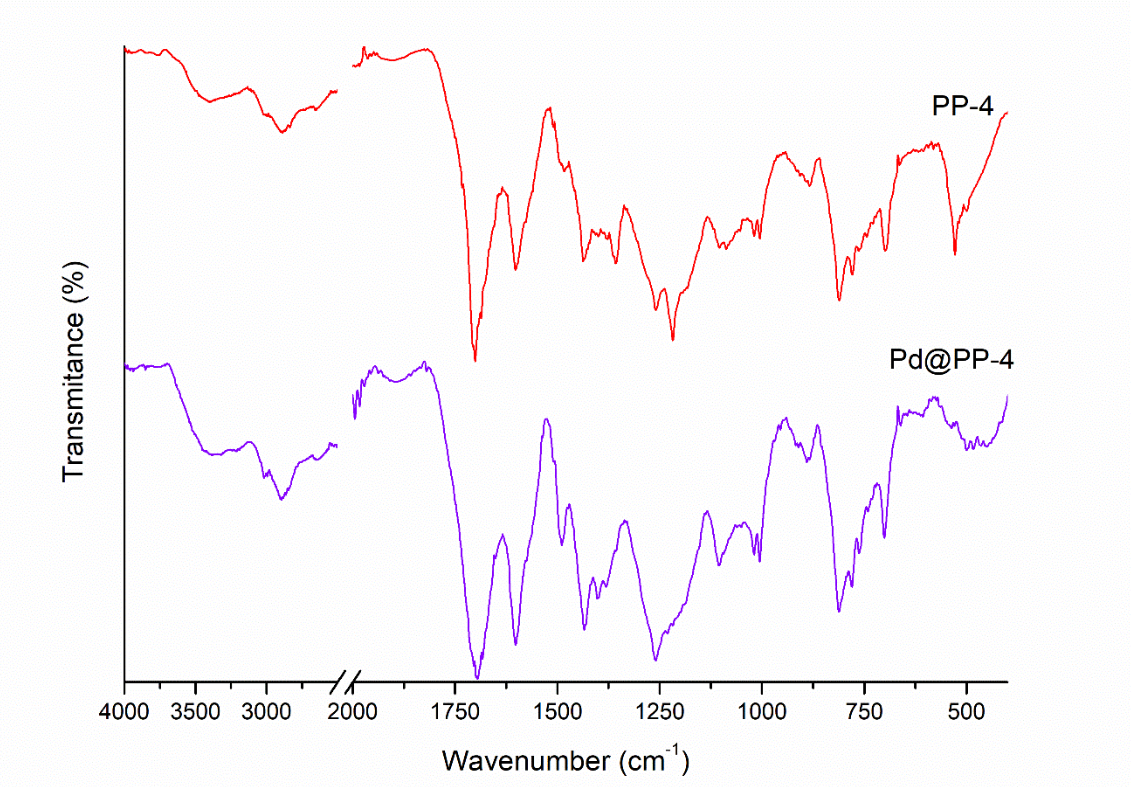

Figure S9. ATR-FTIR of Pd@PP-4 and its PP-4 precursor.

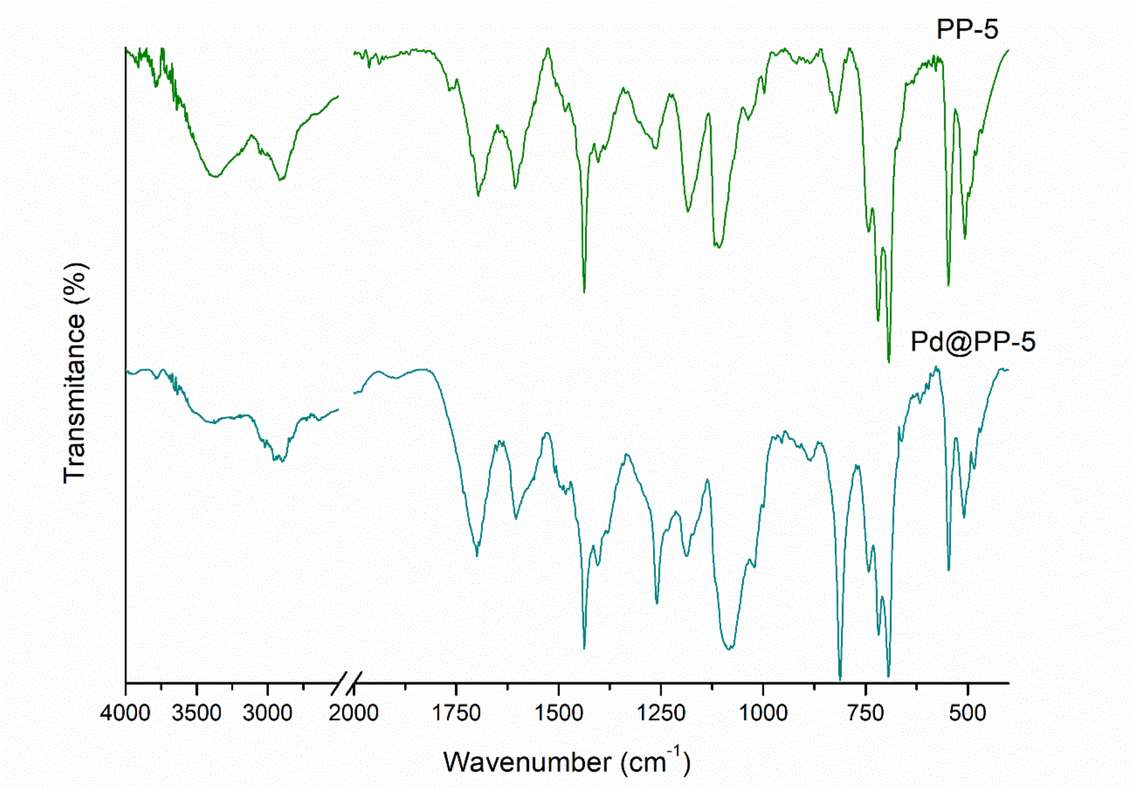

Figure S10. ATR-FTIR of Pd@PP-5 and its PP-5 precursor.

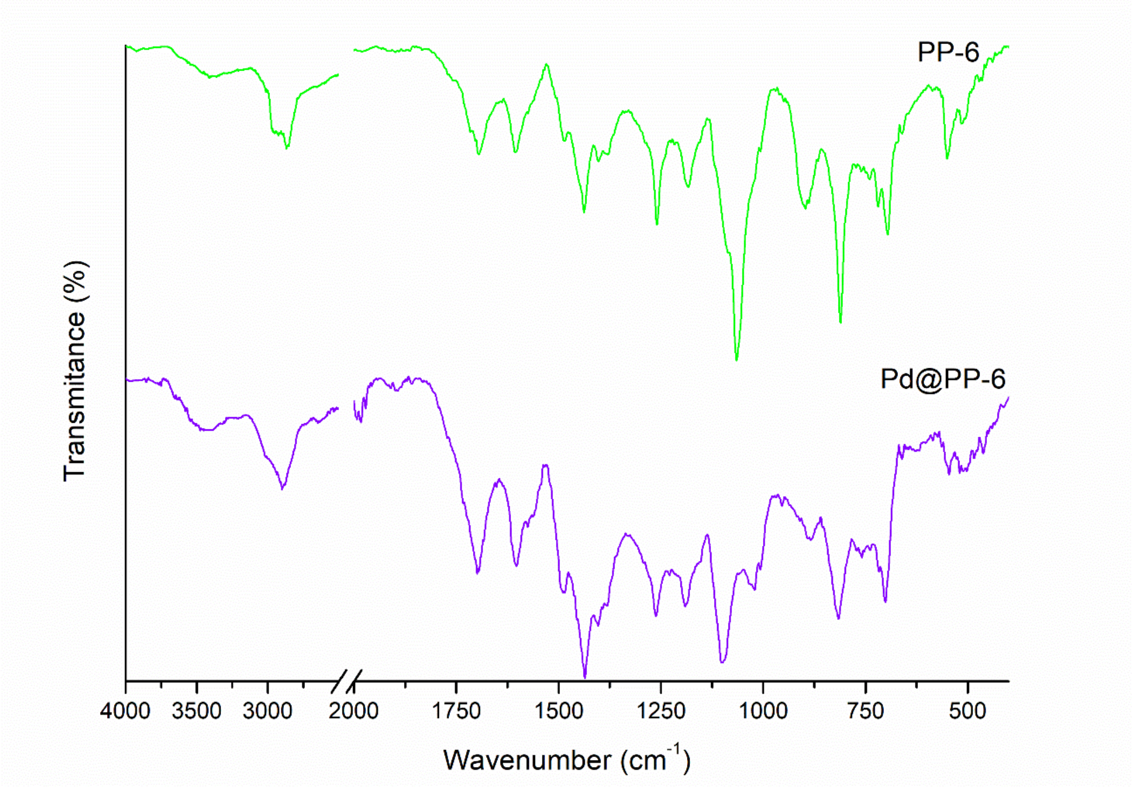

Figure S11. ATR-FTIR of Pd@PP-6 and its PP-6 precursor.

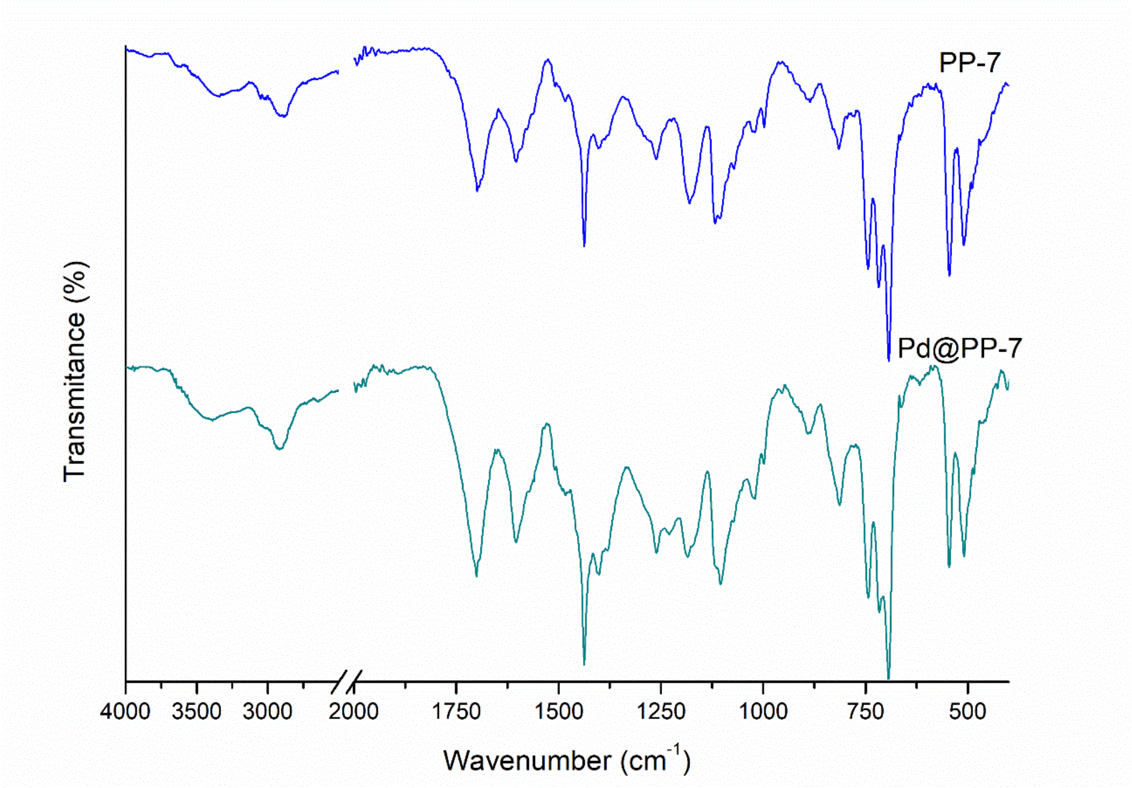

Figure S12. ATR-FTIR of Pd@PP-7 and its PP-7 precursor.

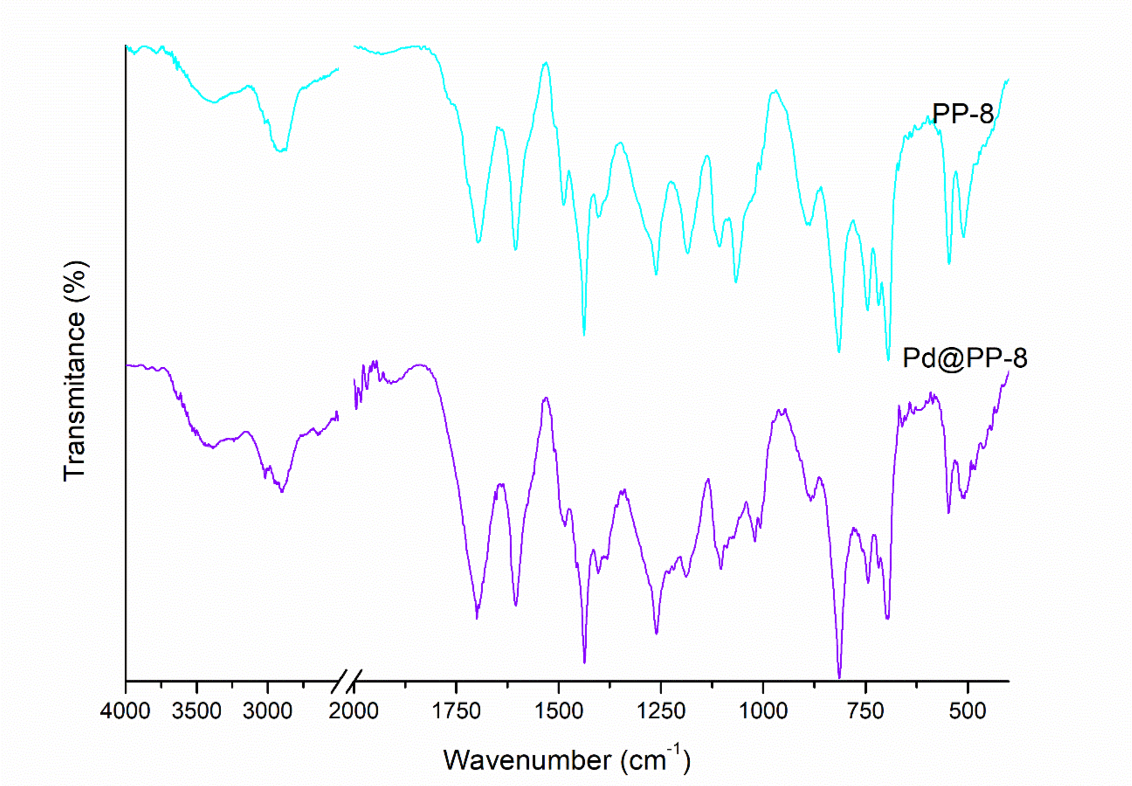

Figure S13. ATR-FTIR of Pd@PP-8 and its PP-8 precursor.

polymers

MDPI

Section 3.2. CPMAS Solid-State NMR of polymer networks

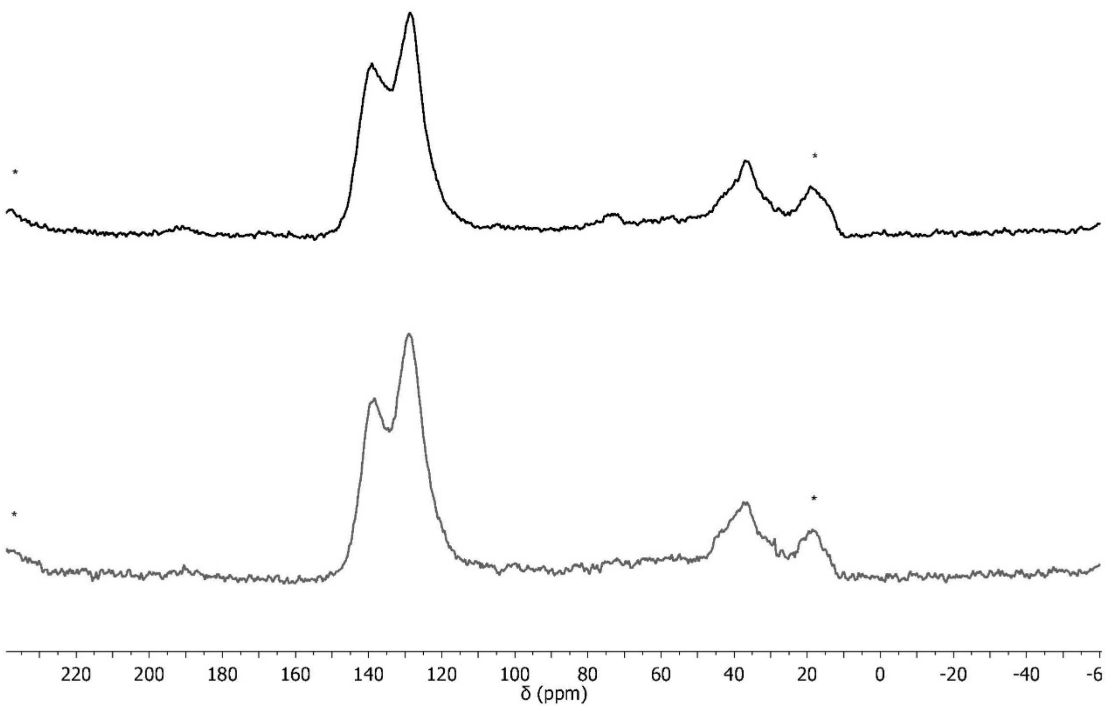

Figure S14. CP/MAS ^{13}C NMR spectra of PP-1 and PP-2 obtained from TPP.

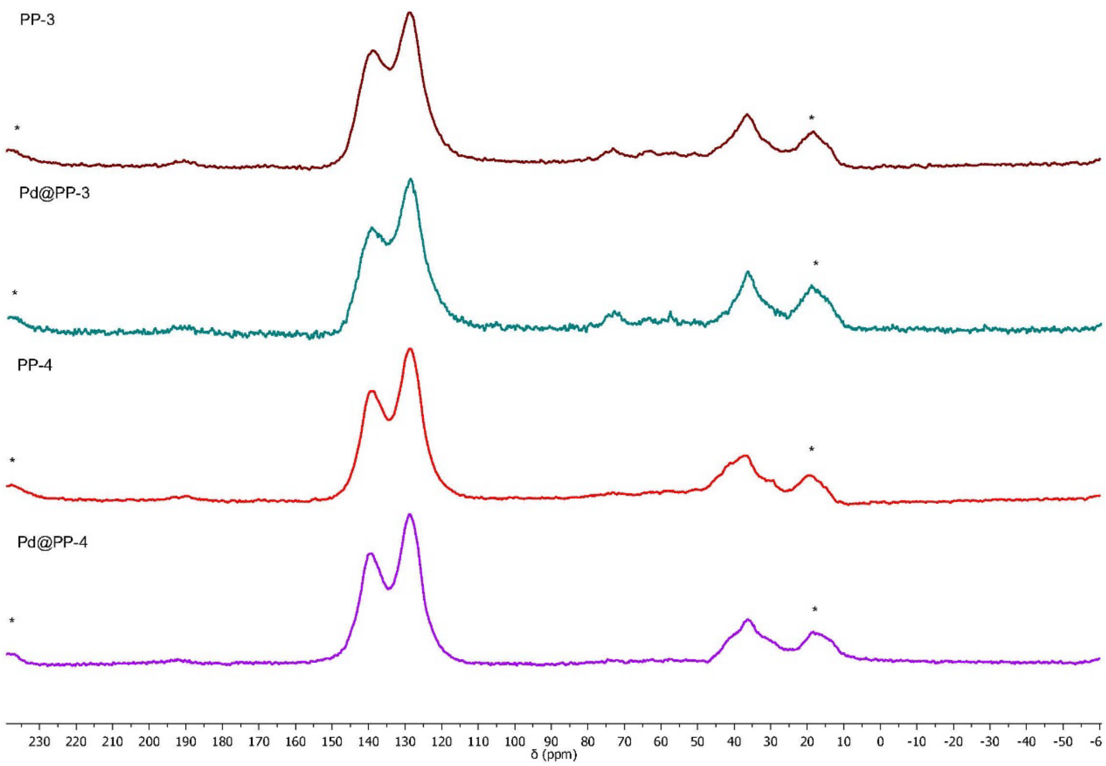

Figure S15. CP/MAS ^{13}C NMR spectra of PP-supports and Pd catalysts obtained from DPPE.

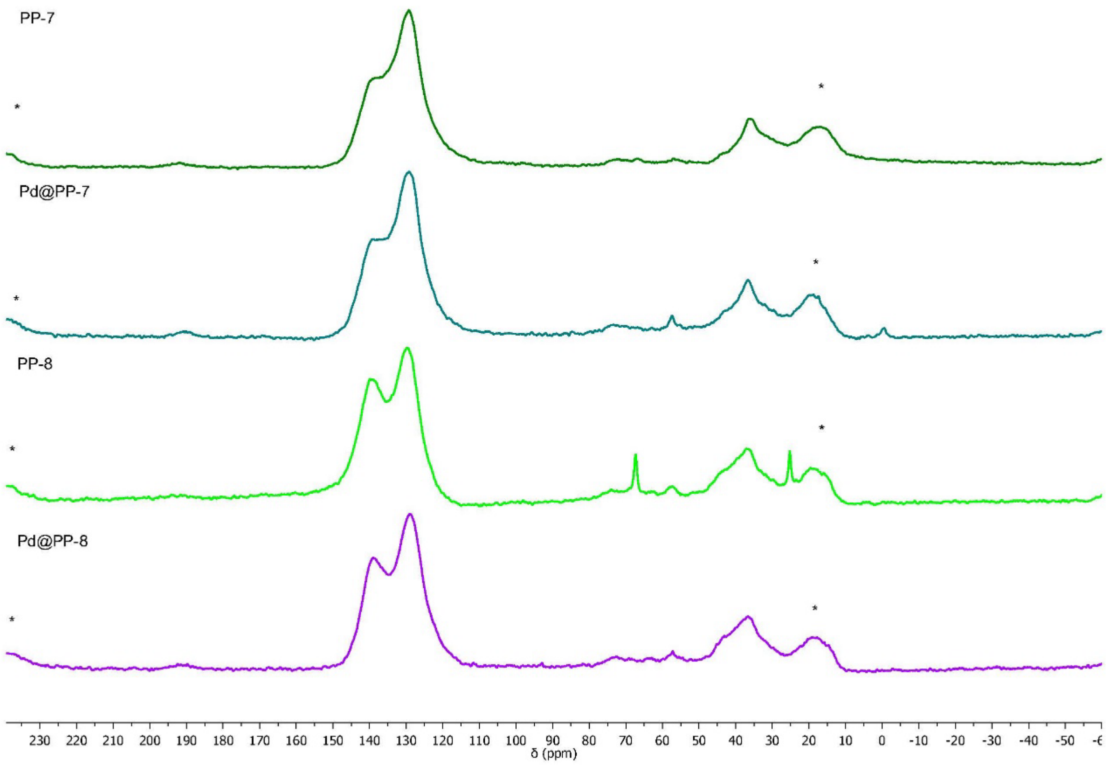

Figure S16. CP/MAS ^{13}C NMR spectra of PP-supports and Pd catalysts obtained from DPPP.

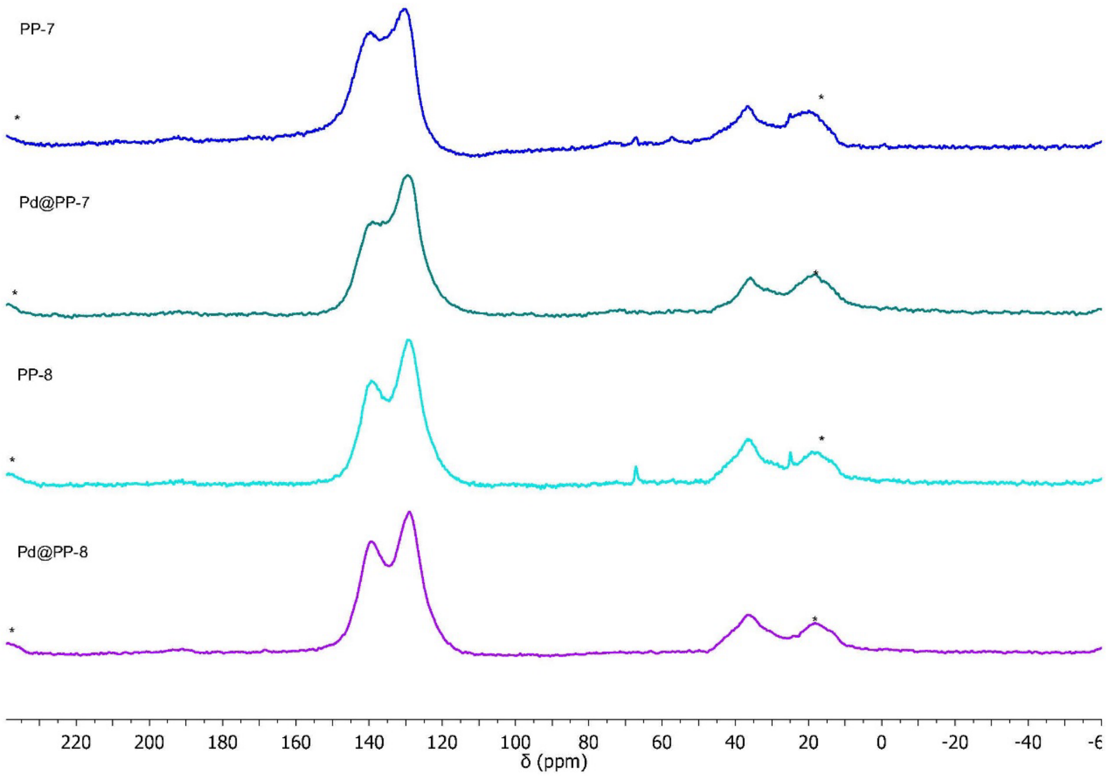

Figure S17. CP/MAS ^{13}C NMR spectra of PP-supports and Pd catalysts obtained from DPPB.

The signal centered at 18 ppm is overlapped with the side-band signal (labeled with *).

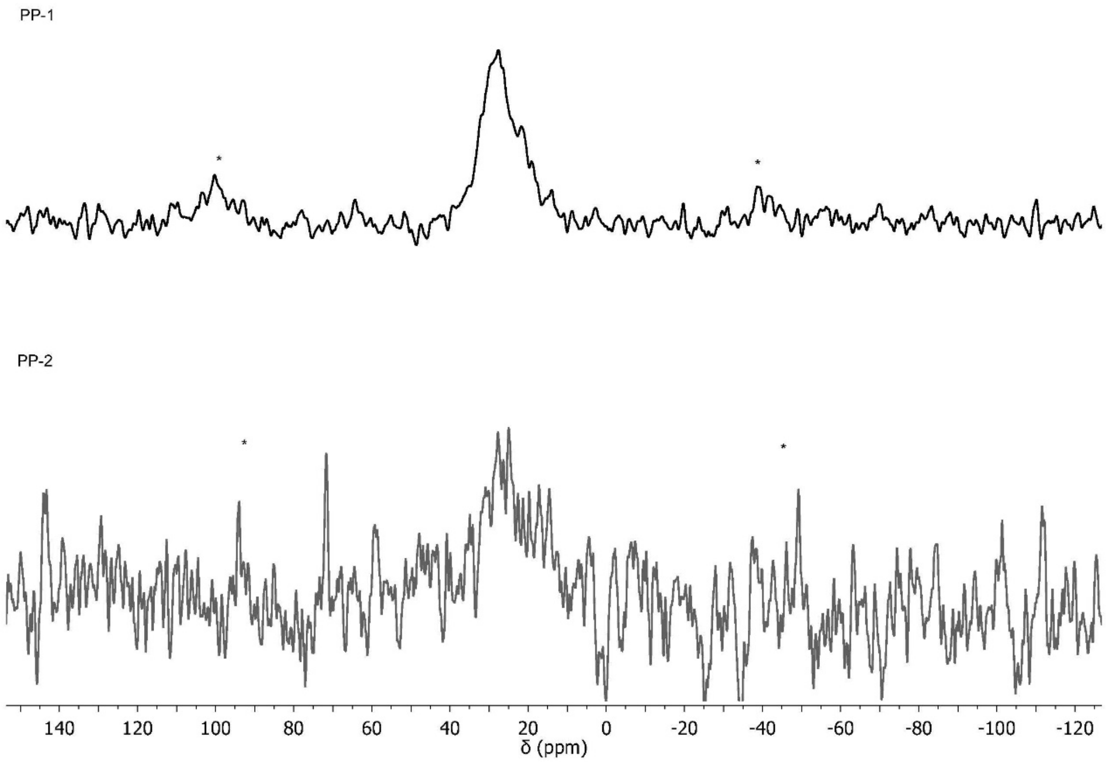

Figure S18. CP/MAS ^{31}P NMR spectra of PP-1 and PP-2 obtained from TPP.

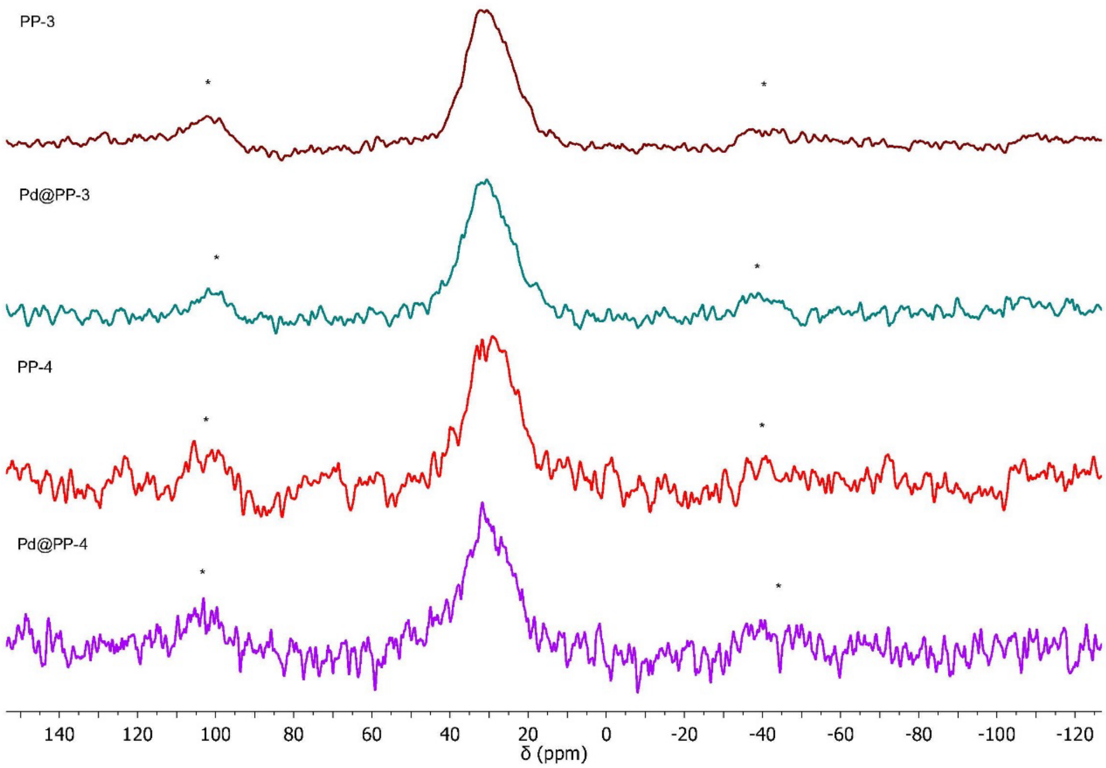

Figure S19. CP/MAS ^{31}P NMR spectra of PP-supports and Pd catalysts obtained from DPPE.

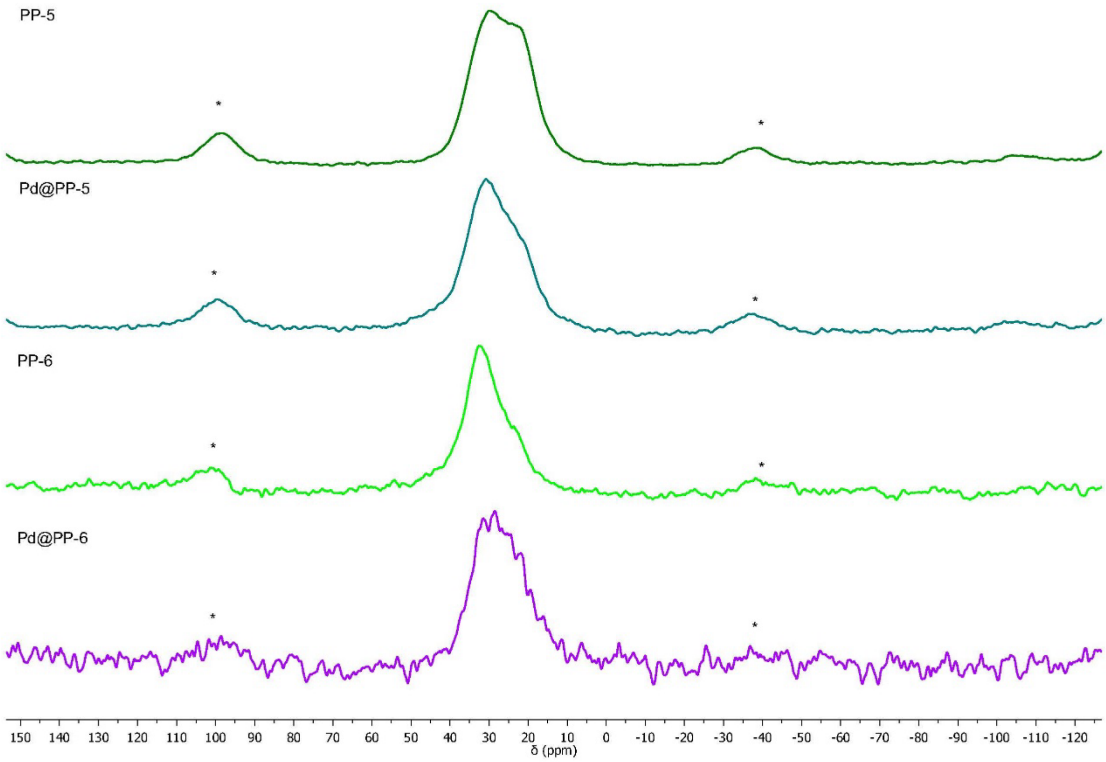

Figure S20. CP/MAS ^{31}P NMR spectra of PP-supports and Pd catalysts obtained from DPPP.

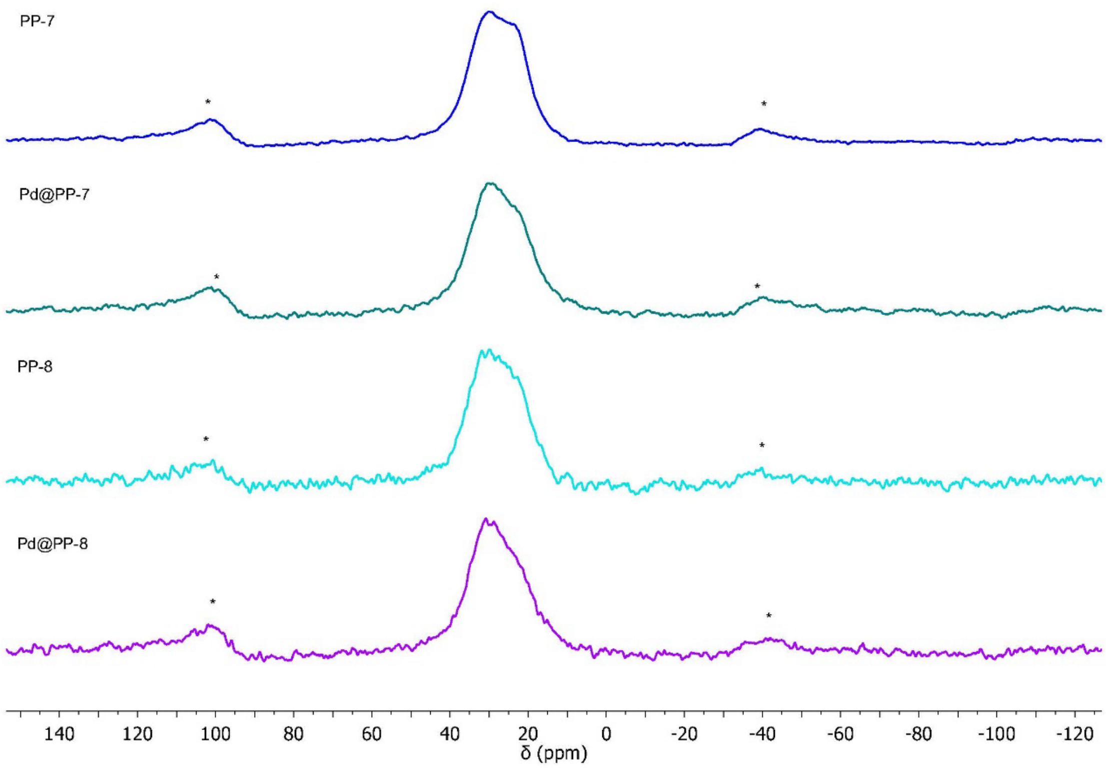

Figure S21.- P/MAS ^{31}P NMR spectra of PP-supports and Pd catalysts obtained from DPPB.

Section 3.3. WAXS of polymer networks

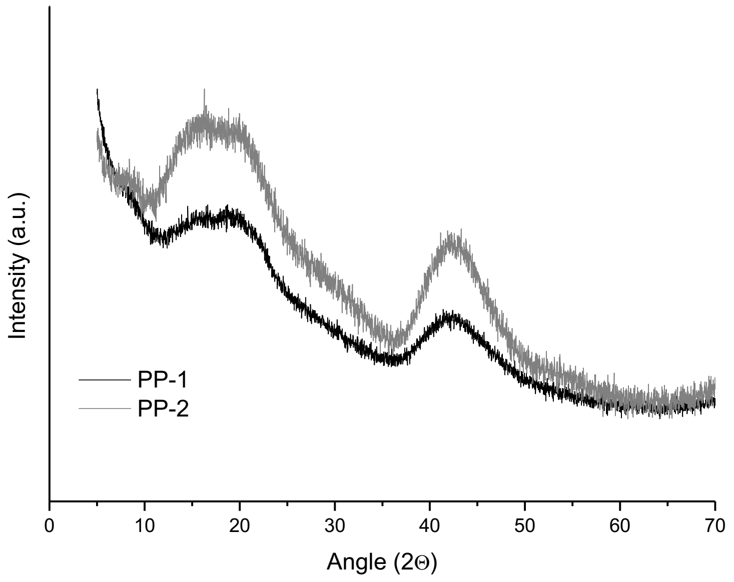

Figure S22. WAXS pattern of PP-1 and PP-2.

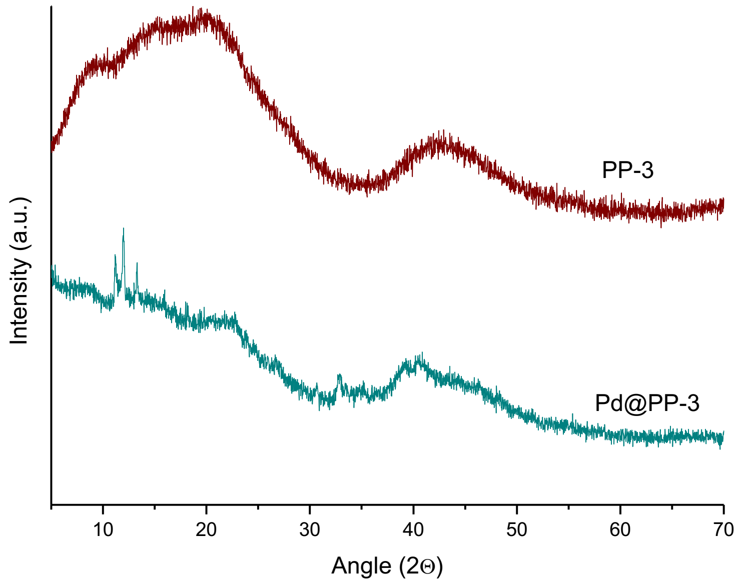

Figure S23. WAXS pattern of Pd@PP-3 and its PP-3 precursor.

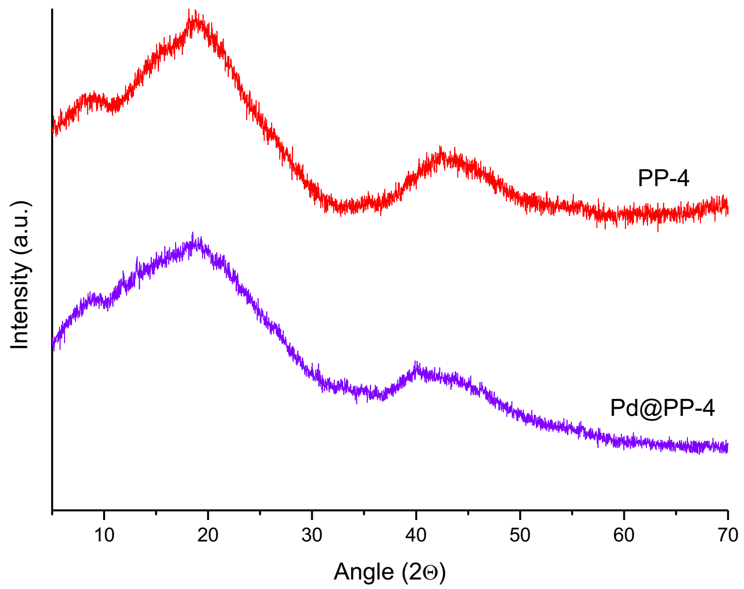

Figure S24. WAXS pattern of Pd@PP-4 and its PP-4 precursor.

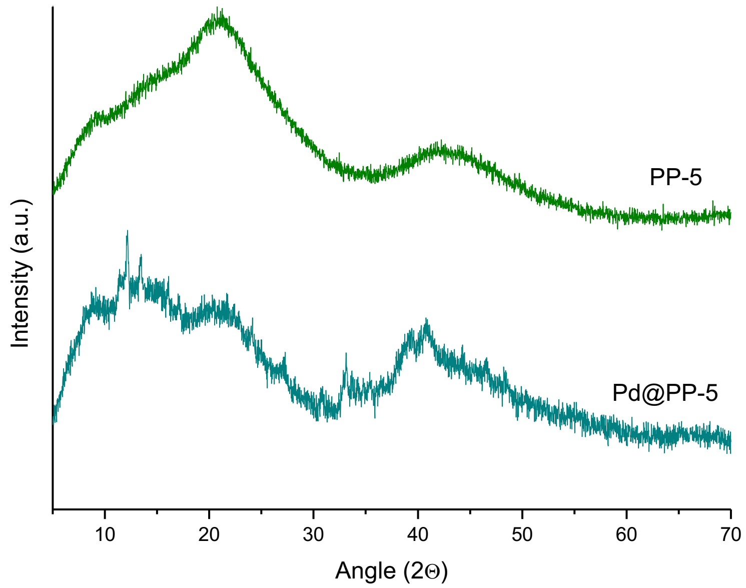

Figure S25. WAXS pattern of Pd@PP-5 and its PP-5 precursor.

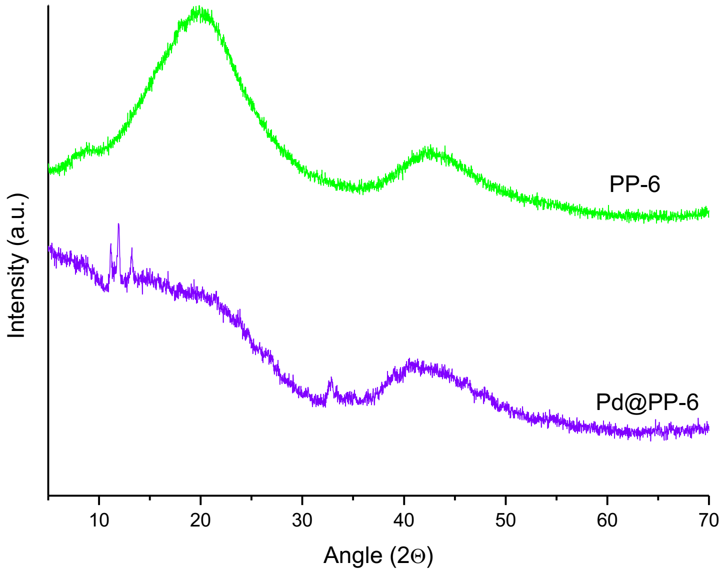

Figure S26. WAXS pattern of Pd@PP-6 and its PP-6 precursor.

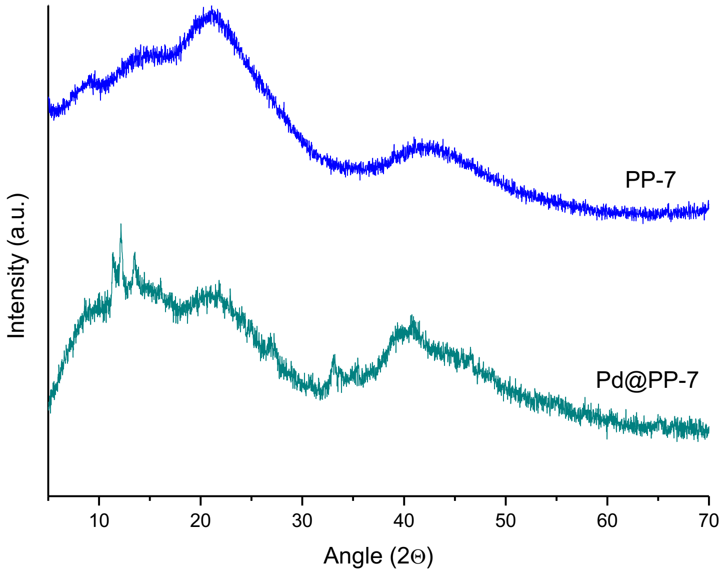

Figure S27. WAXS pattern of Pd@PP-7 and its PP-7 precursor.

polymers

MDPI

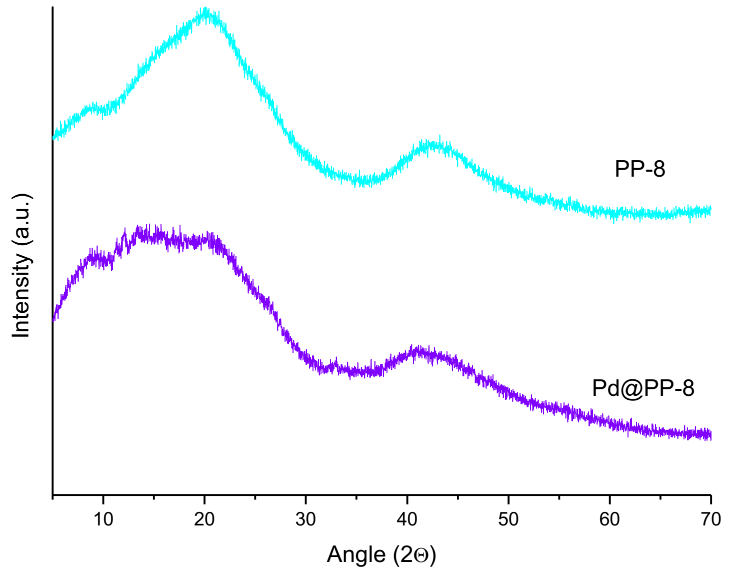

Figure S28. WAXS pattern of Pd@PP-8 and its PP-8 precursor.

Section 3.4. Dynamic thermogravimetric analysis (TGA) of polymer networks

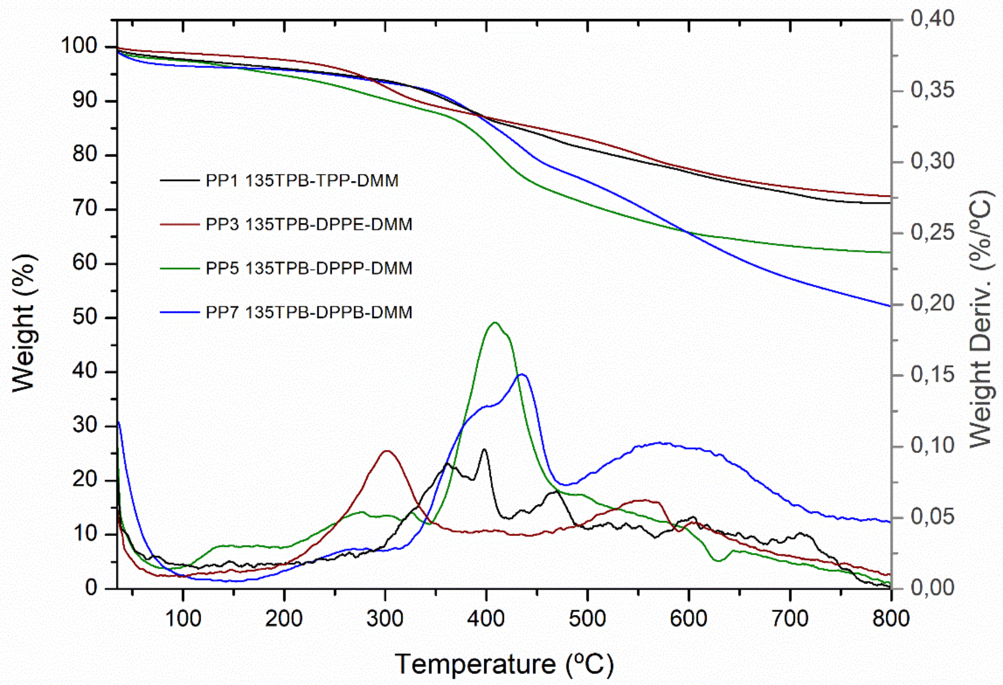

Figure S29. Thermograms of phosphine-POPs without BP.

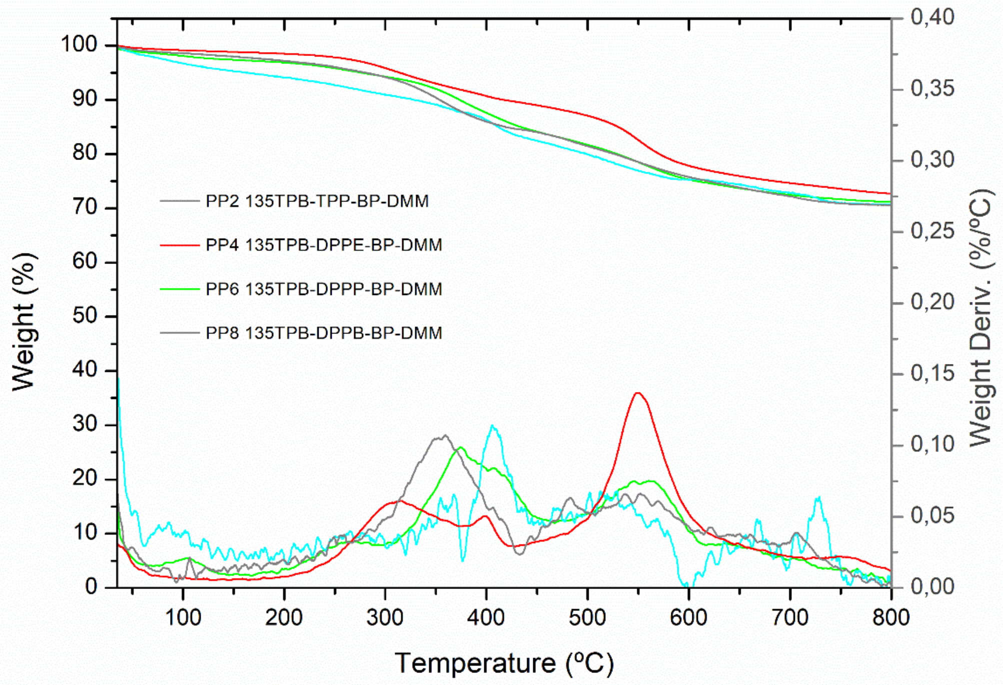

Figure S30. Thermograms of phosphine-POPs with BP.

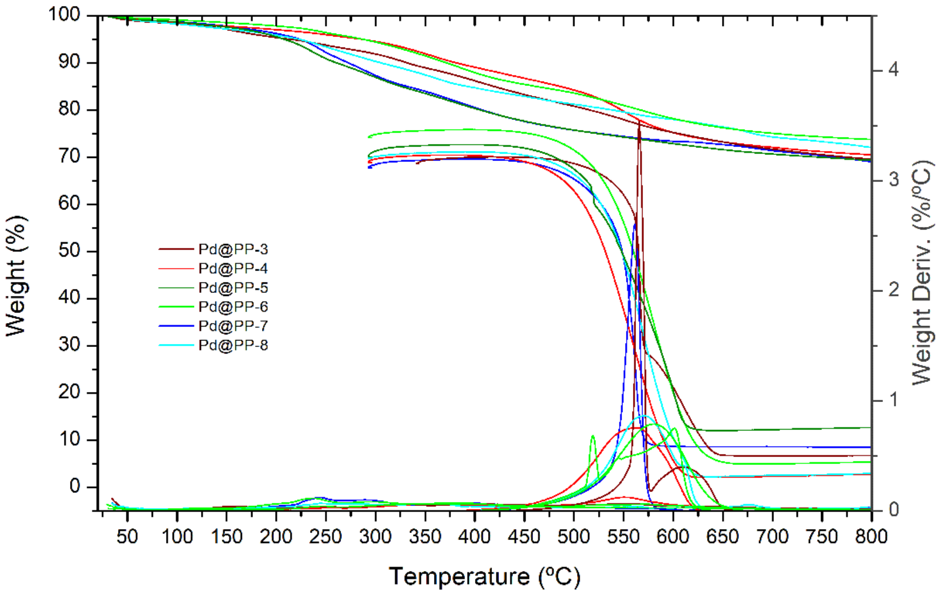

Figure S31. Thermograms of phosphine-POPs-Pd(II) catalysts.

Section 3.5. Gas adsorption isotherms and pore distribution of polymer networks

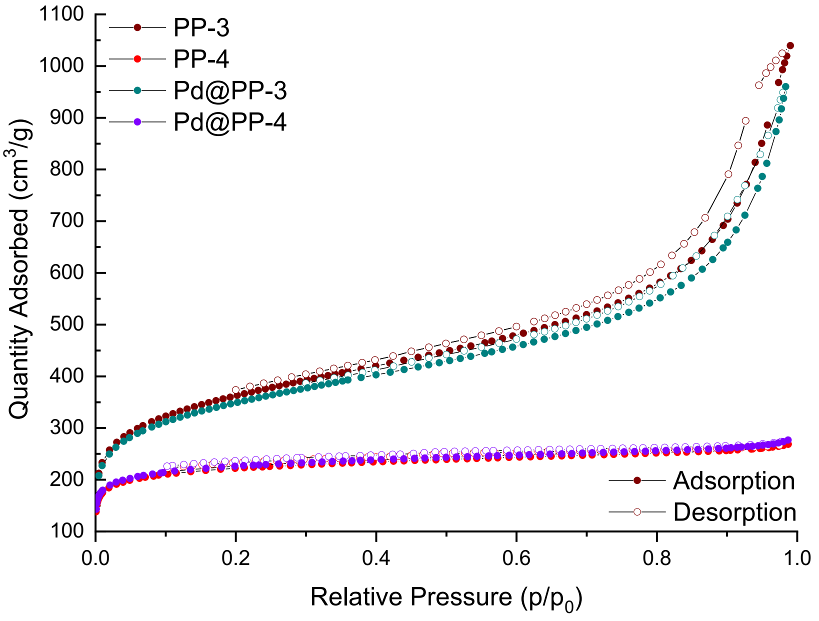

Figure S32. N_2 adsorption (full symbols)/desorption (empty symbols) isotherms of polymers synthetized from DPPE measured at 77 K.

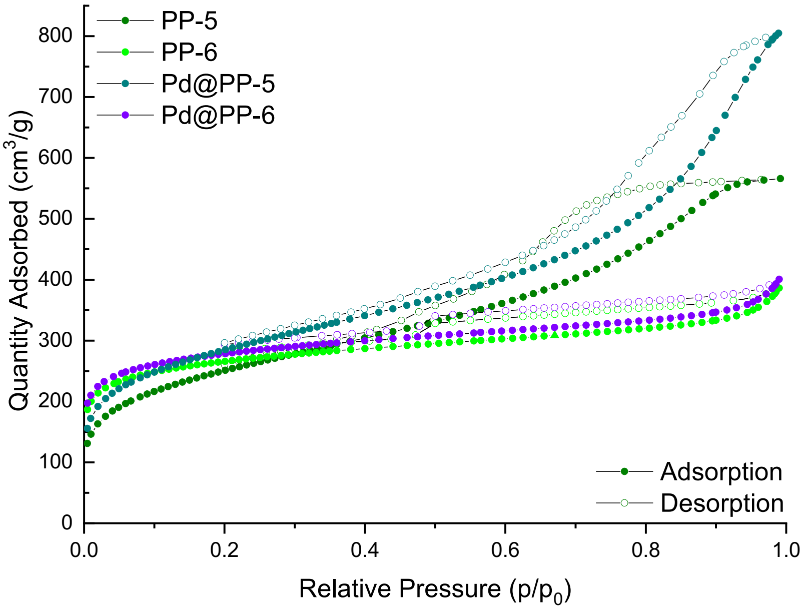

Figure S33. N_2 adsorption (full symbols)/desorption (empty symbols) isotherms of polymers synthetized with DPPP measured at 77 K.

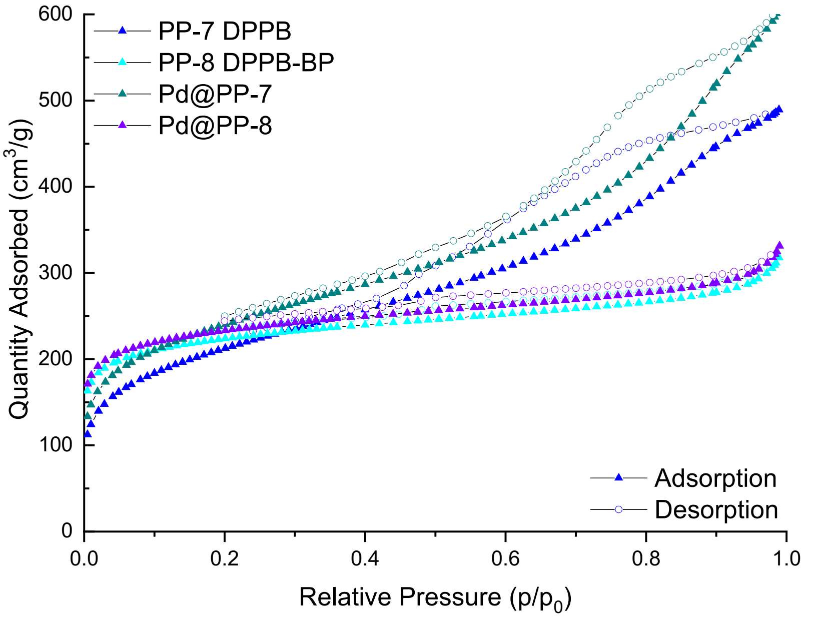

Figure S34. N_2 adsorption (full symbols)/desorption (empty symbols) isotherms of polymers synthetized with DPPB measured at 77 K.

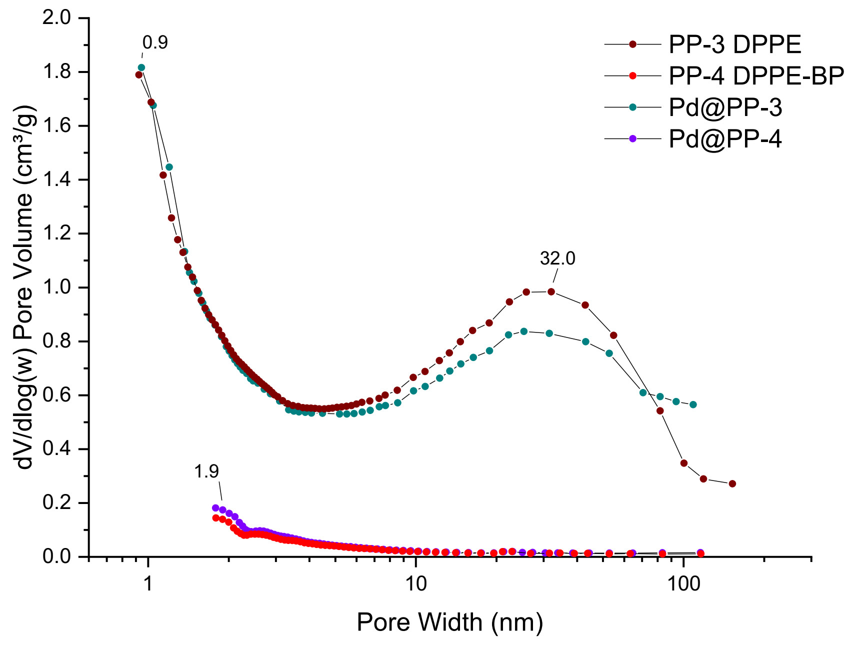

Figure S35. Adsorption porous distribution of network polymers obtained from DPPE.

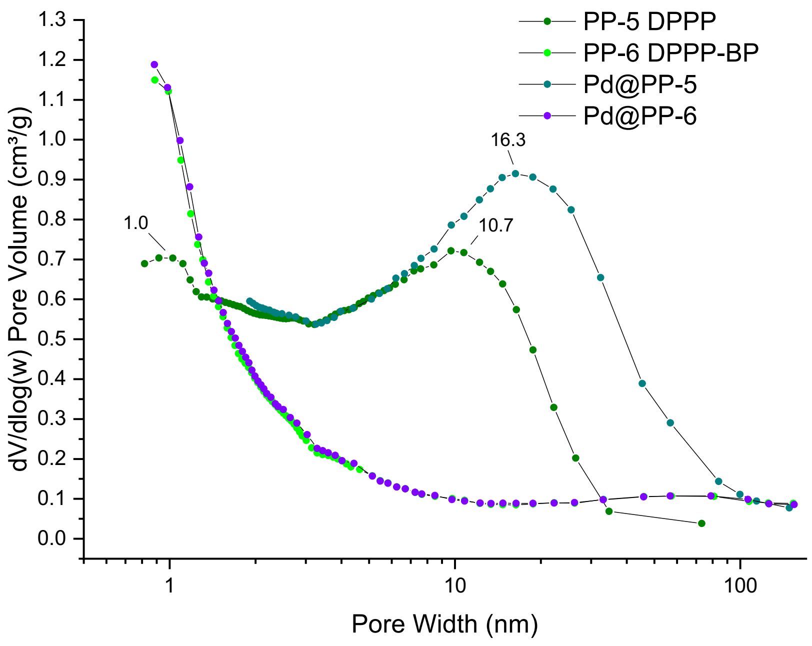

Figure S36. Adsorption porous distribution of network polymers obtained from DPPP.

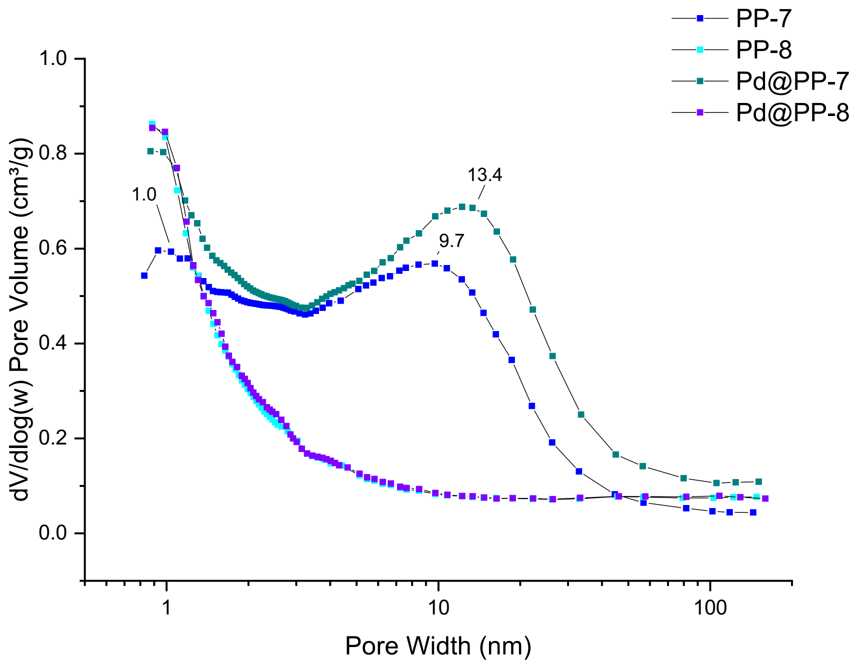

Figure S37. Adsorption porous distribution of network polymers obtained from DPPB.

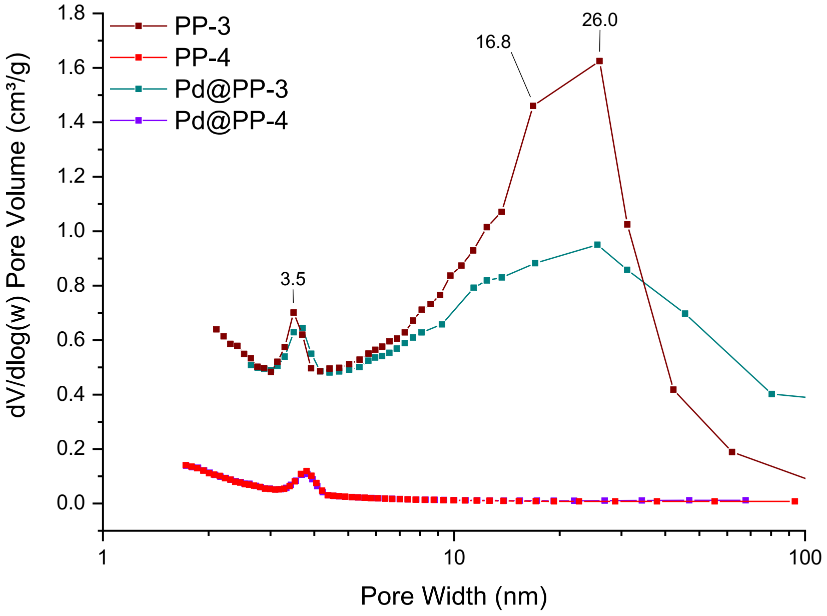

Figure S 38. Desorption porous distribution of network polymers obtained from DPPE.

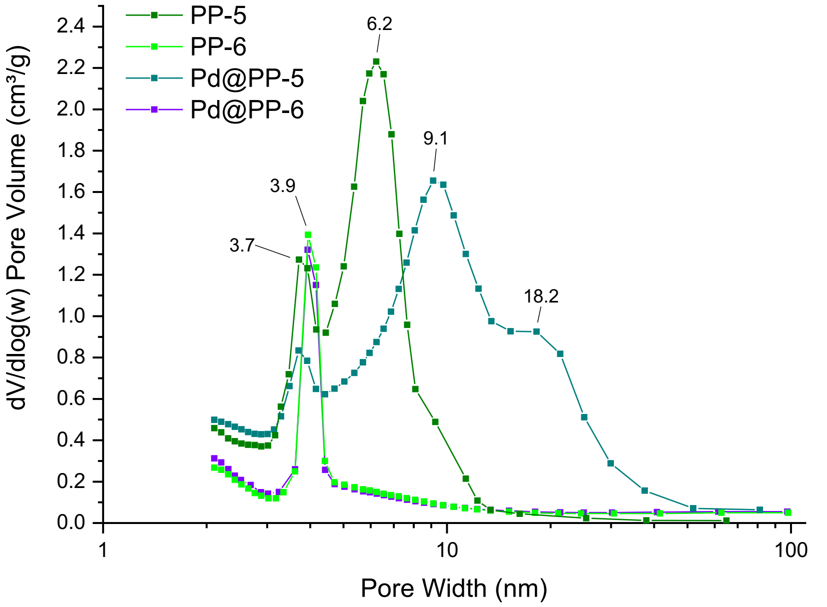

Figure S39. Desorption porous distribution of network polymers obtained from DPPP.

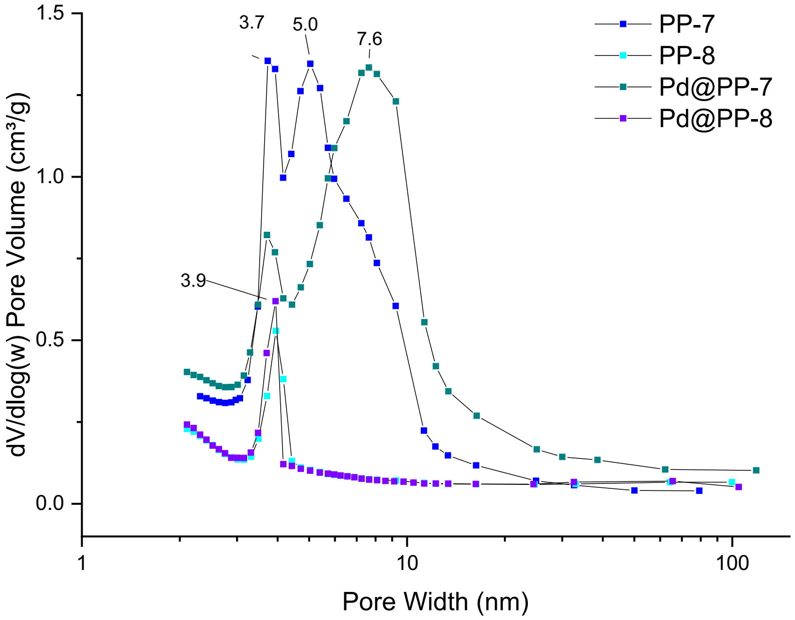

Figure S40. Desorption porous distribution of network polymers obtained from DPPB.

Table S1. Porosity Parameters derived from N_2 Adsorption Isotherms

		BET (m^2/g)	Pore size ad/des (nm)	V total by BJH (cm^3/g)	V μpore	V meso + macro	% micropore
PP-3	135TPB-DPPE-DMM (4:3:12)	1302	4.72/.1.78	1.54	0.26	1.28	16.6
Pd@PP-3	135TPB-DPPE-DMM + 50% $Pd(OAc)_2$	1255	4.42/1.91	1.39	0.29	1.10	20.8
PP-4	135TPB-DPPE-BP-DMM (4:3:12:24)	835	1.99/1.64	0.42	0.28	0.14	67.3
Pd@PP-4	135TPB-DPPE-BP-DMM + 50% $Pd(OAc)_2$	852	2.01/1.64	0.43	0.31	0.11	73.2
PP-5	135TPB-DPPP-DMM (4:3:12)	896	0.91/3.91	0.88	0.12	0.76	13.5
Pd@PP-5	135TPB-DPPP-DMM + 50% $Pd(OAc)_2$	1015	4.84/1.81	1.23	0.20	1.03	15.9
PP-6	135TPB-DPPP-BP-DMM (4:3:12:24)	990	2.33/1.75	0.58	0.30	0.28	52.0
Pd@PP-6	135TPB-DPPP-BP-DMM + 50% $Pd(OAc)_2$	1038	2.3/1.75	0.60	0.36	0.23	60.9
PP-7	135TPB-DPPB-DMM (4:3:12)	767	3.9/1.9	0.75	0.12	0.63	16.4
Pd@PP-7	135TPB-DPPB-DMM + 50% $Pd(OAc)_2$	862	4.2/1.7	0.92	0.15	0.76	16.7
PP-8	135TPB-DPPB-BP-DMM (4:3:12:24)	839	2.2/1.7	0.47	0.28	0.19	60.1
Pd@PP-8	135TPB-DPPB-BP-DMM + 50% $Pd(OAc)_2$	875	2.25/1.7	0.49	0.30	0.19	60.5

Section 3.6. SEM and SEM-EDX characterization of Pd-supported catalysts

Samples of POPs and Pd-POPs were prepared as follow: A suspension of 5 mg of polymer in 2 mL of EtOH was sonicated for 10 min at 25% of amplitude (2 s on, 1 s off) to disperse the particles. After that, the solution was sonicated at 30% of amplitude (2 s on, 1 s off) for 2 min and 0.3 mL of the central zone of the suspension was transferred to another vial and diluted with EtOH until 2 mL of volume. The diluted sample was sonicated at 50% of amplitude for 30 s (1 s on, 1 s off) and a drop of the central zone of the solution was deposed in a carbon film. In the case of POPs, the samples were metalized with Au for 15 s (~1 nm thickness).

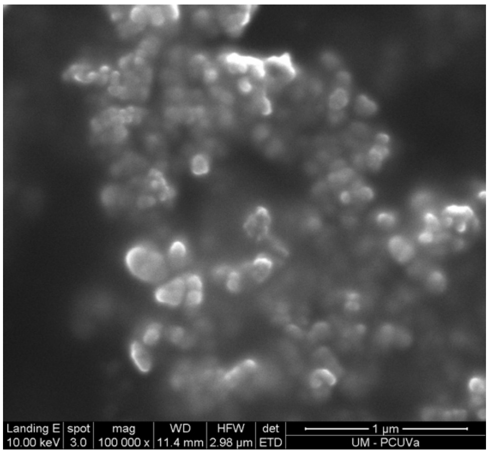

Figure S41. SEM image of PP-3.

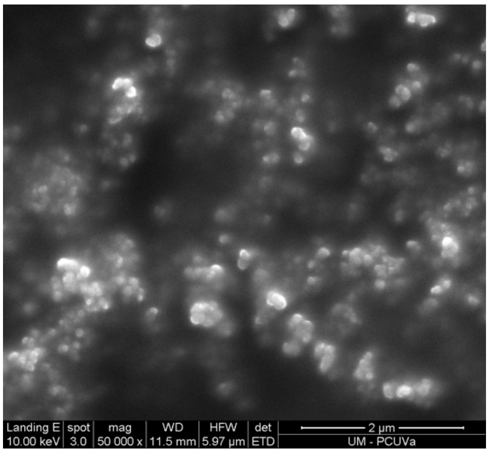

Figure S42. SEM image of PP-4

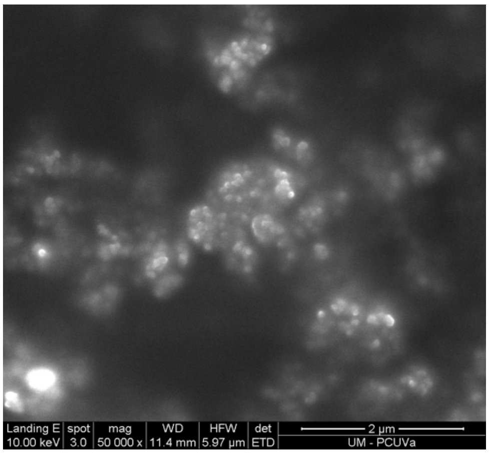

Figure S43. SEM image of PP-5.

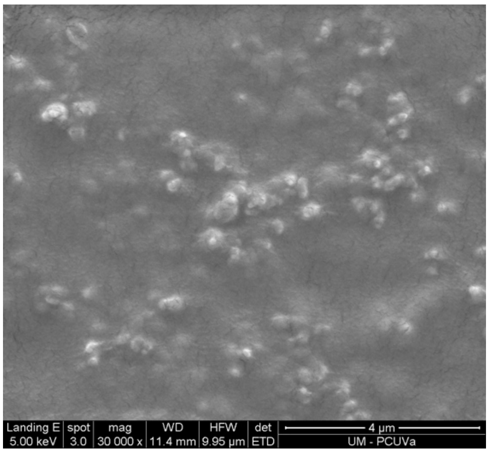

Figure S44. SEM image of PP-6.

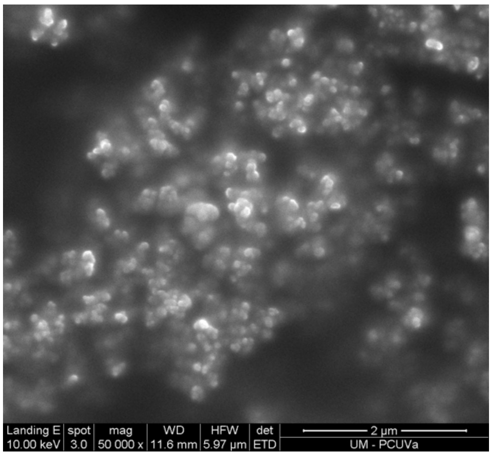

Figure S45. SEM image of PP-7.

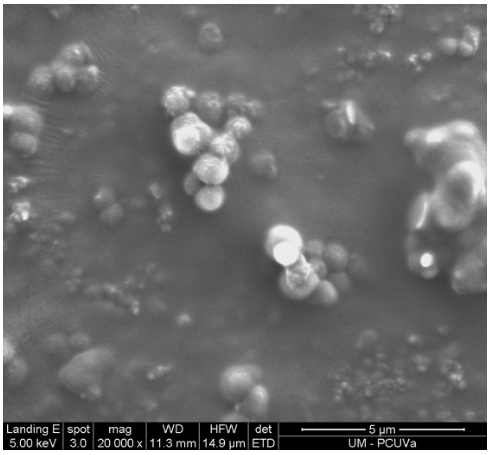

Figure S46. SEM image of PP-8.

The samples of Pd@POPs for SEM EDAX were deposed as powders on a carbon film.

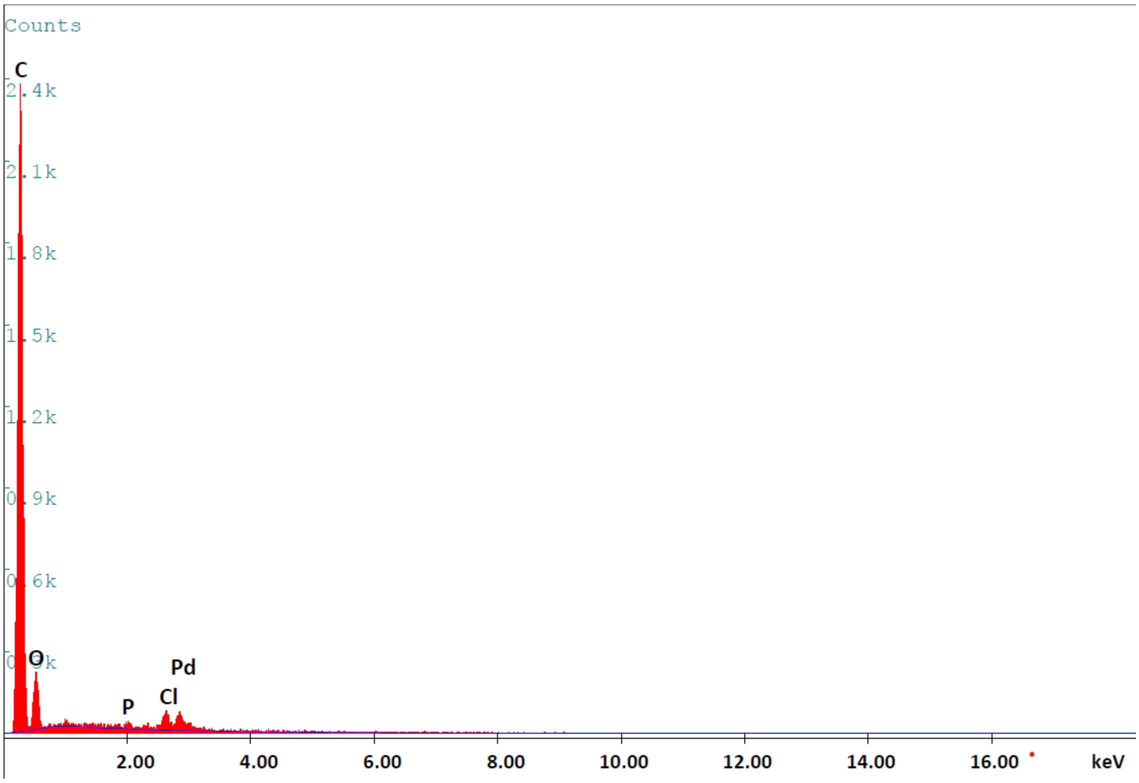

Figure S47. SEM-EDX of Pd@PP-3.

Section 3.7. XPS of Pd catalysts

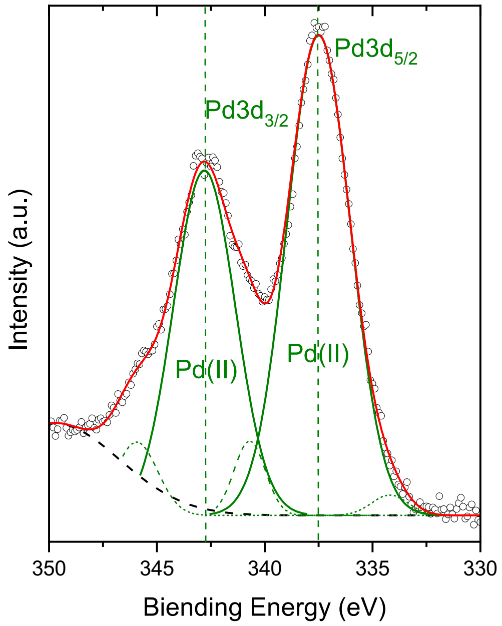

Figure S48. XPS analysis of Pd3d binding energies for Pd@PP3.

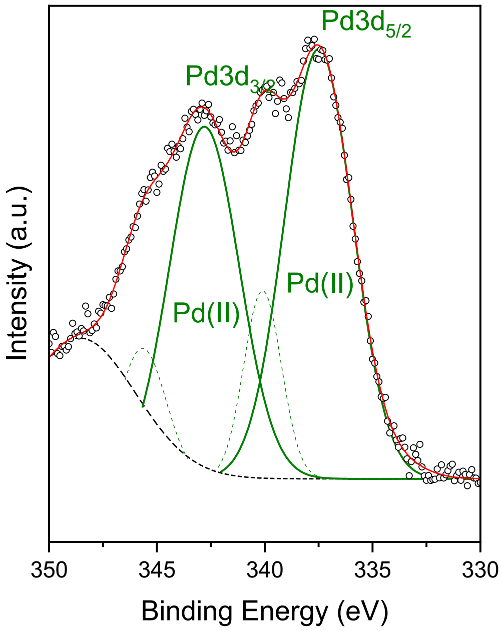

Figure S49. XPS analysis of Pd3d binding energies for Pd@PP4.

Section 4. Catalytic activity

Table S2-Leaching test

	Time	Conversion (%)
1 mol% of Pd@PP-3	5 min	77
No catalyst	+ 15 min	78
No catalyst	+ 30 min	78

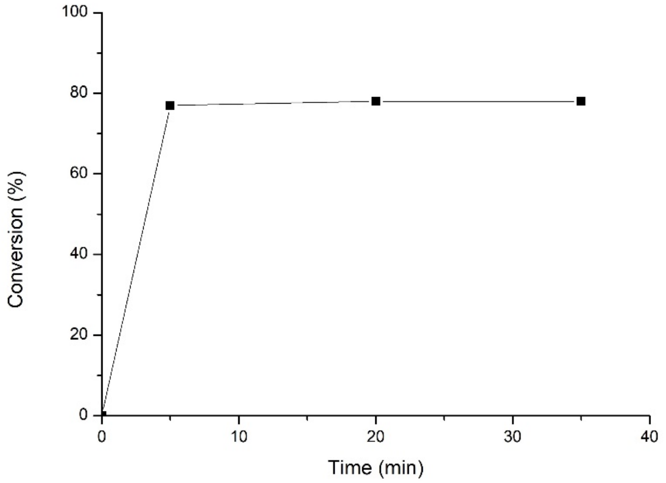

Figure S50. Leaching test of Pd@PP-3.
